# Considerations for modelling diffuse high-grade gliomas and developing clinically relevant therapies

**DOI:** 10.1007/s10555-023-10100-7

**Published:** 2023-04-01

**Authors:** Sarah L. Higginbottom, Eva Tomaskovic-Crook, Jeremy M. Crook

**Affiliations:** 1 Intelligent Polymer Research Institute, AIIM Facility, Innovation Campus, University of Wollongong, Fairy Meadow, NSW 2519 Australia; 2 Arto Hardy Family Biomedical Innovation Hub, Chris O’Brien Lifehouse, Camperdown, NSW 2050 Australia; 3 School of Medical Sciences, Faculty of Medicine and Health, The University of Sydney, Camperdown, NSW 2006 Australia

**Keywords:** High-grade glioma, Tumour microenvironment, Glioma stem cells, Clinically relevant, Preclinical models, Organoids, Tissue engineering, 3D printing, Microfluidics

## Abstract

Diffuse high-grade gliomas contain some of the most dangerous human cancers that lack curative treatment options. The recent molecular stratification of gliomas by the World Health Organisation in 2021 is expected to improve outcomes for patients in neuro-oncology through the development of treatments targeted to specific tumour types. Despite this promise, research is hindered by the lack of preclinical modelling platforms capable of recapitulating the heterogeneity and cellular phenotypes of tumours residing in their native human brain microenvironment. The microenvironment provides cues to subsets of glioma cells that influence proliferation, survival, and gene expression, thus altering susceptibility to therapeutic intervention. As such, conventional *in vitro* cellular models poorly reflect the varied responses to chemotherapy and radiotherapy seen in these diverse cellular states that differ in transcriptional profile and differentiation status. In an effort to improve the relevance of traditional modelling platforms, recent attention has focused on human pluripotent stem cell-based and tissue engineering techniques, such as three-dimensional (3D) bioprinting and microfluidic devices. The proper application of these exciting new technologies with consideration of tumour heterogeneity and microenvironmental interactions holds potential to develop more applicable models and clinically relevant therapies. In doing so, we will have a better chance of translating preclinical research findings to patient populations, thereby addressing the current derisory oncology clinical trial success rate.

## Introduction

Diffuse high-grade gliomas (HGGs) are a highly heterogeneous group of tumours that contain some of the most dangerous human cancers [1]. Like other gliomas, HGGs arise from glial or glial precursor cells and are the most common group of malignant central nervous system (CNS) tumours in adults and children [1]. CNS World Health Organisation (WHO) grades 1–4 are assigned to tumours, with a higher grade corresponding to a more aggressive cancer [2]. Current treatments for HGGs (grades 3 and 4) are typically not curative and involve surgical resection, where possible, followed by radiotherapy with concurrent and adjuvant chemotherapy [3–5]. Despite this aggressive treatment and decades of research, patients almost always experience tumour recurrence, with no substantial improvements in outcome [4, 6–8].

In 2021, the WHO revised the classification of gliomas to place greater emphasis on underlying molecular aetiologies, with tumour grading no longer based solely on histology and adult and paediatric forms of glioma recognised as separate disease types [2]. The 2021 WHO classification of tumours of the CNS divides gliomas into adult-type diffuse gliomas, paediatric-type diffuse low-grade gliomas (LGGs, grades 1 and 2), paediatric-type diffuse HGGs (grades 3 and 4), circumscribed astrocytic gliomas and ependymomas [2]. Diffuse HGGs readily infiltrate the surrounding brain parenchyma and display a high degree of intertumoural and intratumoural heterogeneity at genetic, epigenetic and microenvironmental levels, presenting major challenges to treatment and therapeutic development [9–17]. Further complexity surrounds the presence of subpopulations of glioma stem cells (GSCs) known to play important roles in tumour initiation, maintenance, and recurrence, with demonstrated resistance to chemoradiotherapy [18–26].

The ability of preclinical models to recapitulate the breadth and plasticity of cellular states present in HGG tumours is critical for the development of clinically relevant therapies. Estimates place the success rate for oncology clinical trials at just 3.4%, indicative of a strong disparity between preclinical models and patient response [27]. Patient stratification based on identified biomarkers increases the clinical trial success rate to 10.7%, underpinning the recent WHO molecular-based classification to improve patient outcomes in neuro-oncology [27]. Although an improvement, this success rate is still low, given that clinical trials in non-oncology fields have an overall success rate of 20.9% [27]. Recent attention has thus focused on the strong contribution of the tumour microenvironment to the maintenance of cellular states and response to therapies [17, 26, 28–30]. The brain microenvironment contains a variety of cell types including neurons, astrocytes, vascular cells, and immune cells that provide distinct survival and proliferation cues to subsets of HGG cells, which may influence treatment outcome [30–33]. Investigating the effects of novel therapies in the absence of these crucial cell-cell interactions or in a non-human host thus limits the relevance and translatability of findings from these preclinical models to patient populations.

Despite differences in disease aetiologies, similar microenvironmental features and stem-like compartments in HGGs prompt similar modelling considerations to interrogate the effects of more targeted treatments. The development of models that more closely reflect the biology of HGG cells in their native microenvironment could thus yield greater success in clinical trials. Recent implementation of human cerebral organoids to model glioblastoma, isocitrate dehydrogenase (IDH)-wildtype tumours produced tumour gene expression signatures that aligned more closely with the original patient tumour than could be achieved by traditional cell culture models or patient-derived xenografts, however, they still failed to recapitulate the full complexity of patient tumours [34]. 3D bioprinting and microfluidic culture techniques have also been applied to HGG modelling to overcome disadvantages associated with traditional culture methods and have shown responses to investigated therapeutics closer to patient populations than standard *in vitro* culture [35, 36]. However, no current model is without limitations and despite significant advances being made, further work in improving tumour modelling platforms is imperative to the study of these devastating diseases. This review therefore considers the heterogeneity and plasticity of HGGs influenced by microenvironmental interactions that are critical to the accuracy of preclinical models, with implications for therapeutic efficacy, and highlights recent advances in tissue engineering techniques that strive to improve relevance.

## Tumour heterogeneity and microenvironmental interactions affect therapeutic response

Recapitulating underlying tumour biology is important to the design and implementation of modelling platforms in which novel therapeutics are investigated if study outcomes are to be relevant. Although differences in response to therapeutic intervention based on WHO classification as well as between patients of the same tumour type exist, HGG tumours share features related to clonal evolution, the cancer stem cell (CSC) hypothesis and cell state plasticity that shape tumour architecture and govern treatment response [4, 7, 11, 17, 26, 37–43]. The surrounding tumour microenvironment is not passive and actively shapes tumour architecture, providing specific cues that promote the growth, maintenance and therapeutic resistance of tumour subpopulations [25, 26, 28, 30, 44–46]. This results in the presence of diverse subpopulations within tumours that respond differently to exogenous insults. The design of modelling platforms that mirror as many of these microenvironmental features as possible, to which HGG cells of different tumour types can be applied, would therefore support the maintenance of tumour heterogeneity to better reflect treatment responses seen in patients. Consideration of how and why tumour subpopulations respond differently to therapeutic intervention, as well as the interactions with cells of the surrounding microenvironment that affect cell phenotype, is therefore necessary when designing HGG modelling platforms for clinical relevance.

### Intertumoural and intratumoural heterogeneity affect patient outcome

Molecular stratification of patient populations has determined significant effects of genetic and epigenetic aberrations and tumour transcriptional profiles on diffuse HGG pathobiology, patient outcome and response to therapy. Accordingly, the 2021 WHO classification of CNS tumours recognised adult- and paediatric-type diffuse HGGs as separate disease types based on different underlying molecular aetiologies [2] (Table 1). Notably, the inability to stratify paediatric glioblastoma patients according to transcriptional profiles prominent in adults, as well as the identification of somatic histone mutations largely exclusive to childhood and adolescent HGG tumours, prompted the dissolution of paediatric glioblastoma as a disease entity and the establishment of new paediatric HGG tumour types [11, 47–57]. Such differences in tumour molecular properties have been associated with differences in patient prognosis and response to therapies; significantly longer overall survival times were seen in isocitrate dehydrogenase 1 (*IDH1*)-mutant (*n* = 28) compared to *IDH1*-wild type (*n* = 395) tumours (35.4 vs 13.3 months, *p* < 0.001) analysed in The Cancer Genome Atlas (TCGA) cohort, while the methylation of the O^6^-methylguanine-DNA methyltransferase (*MGMT*) promoter has been heavily implicated in tumour responsiveness to alkylating chemotherapeutic agents temozolomide and carmustine [11, 41–43]. Moreover, despite decisions regarding the treatment of paediatric HGGs to date being largely based on the results of adult clinical trials, amounting evidence suggests that these results may not translate to the paediatric setting, where tumours have markedly different molecular features. Since the landmark phase III clinical trial by Stupp et al. [4] in 2005, the standard of care for newly diagnosed adult grade 4 glioma patients following surgical resection has been concurrent temozolomide (75 mg/m^2^/d for ≤ 7 weeks) and radiotherapy followed by 6–12 cycles of adjuvant temozolomide (150–200 mg/m^2^ on 5-d therapy every 28 days) [3–5]. This regimen was demonstrated to improve the 2-year overall survival to 26.5%, compared to 10.4% for radiotherapy (60 Gy delivered in 30 daily fractions of 2 Gy each) alone, and 13% for previously used chemotherapeutics carmustine and lomustine in a separate trial [4, 58]. Although promising in adult patients, the ACNS0126 single-arm phase II study by the Children’s Oncology Group suggested that temozolomide (3-year event-free survival of 7%) did not produce the same improvement in survival compared to a lomustine, vincristine and prednisone (3-year event-free of 15%) chemotherapy regimen [7, 40]. Thus, the need to tailor studies to specific tumour types in models capable of recapitulating the differences in underlying molecular properties is apparent.

**Table 1:** Properties of diffuse high-grade glioma tumour types

Tumour	Grades	Molecular features	References
Adult-type diffuse gliomas			
Glioblastoma, IDH-wildtype	4	*TERT* promoter mutation, gain/loss of chromosomes 7/10, and/or *EGFR* amplification. IDH-wildtype	[2]
Astrocytoma, IDH-mutant	2–4	Aberrations in *IDH1*, *IDH2*, *ATRX*, *TP53* and/or *CDKN2A/B* (WHO grade 4). G-CIMP phenotype	[2, 11, 59]
Oligodendroglioma, IDH-mutant and 1p/19q-codeleted	2–3	Alterations in *IDH1*, *IDH2*, *CIC*, *FUBP1*, *NOTCH1* and/or *TERT* promoter with chromosome arms 1p and 19q deleted. G-CIMP phenotype	[2, 60]
Paediatric-type diffuse HGGs			
Diffuse paediatric-type HGG, H3-wildtype and IDH-wildtype	4	Aberrations in *PDGFRA*, *EGFR* and/or *MYCN*. IDH- and H3-wildtype	[2]
Diffuse hemispheric glioma, H3 G34-mutant	4	H3 G34 substitution. Mutations in *TP53* and/or *ATRX*	[2, 61]
Diffuse midline glioma, H3 K27-altered	4	H3 K27 alteration. Mutations in *TP53*, *ACVR1*, *PDGFRA*, *EGFR* and/or *EZHIP*	[2, 62]
Infant-type hemispheric glioma	HG	Alterations to NTRK family, *ALK*, *ROS* and/or *MET*	[2, 63, 64]

ACVR1, activin A receptor type 1. ALK, anaplastic lymphoma kinase. ATRX, ATRX chromatin remodeller. CDKN2A/B, cyclin-dependent kinase inhibitor 2A/B. CIC, capicua transcriptional repressor. EGFR, epidermal growth factor receptor. EZHIP, enhancer of zeste homologs inhibitory protein. FUBP1, far upstream element-binding protein 1. G-CIMP, glioma CpG island methylator phenotype. HG, high-grade. IDH, isocitrate dehydrogenase. MET, mesenchymal epithelial transition proto-oncogene receptor tyrosine kinase. NTRK, neurotrophic tyrosine kinase receptor. PDGFRA, platelet-derived growth factor receptor-α. TERT, telomerase reverse transcriptase. TP53, tumour protein p53. WHO, World Health Organisation

Proneural, mesenchymal and classical transcriptional subtypes of glioblastoma, IDH-wildtype tumours have been demonstrated to affect the therapeutic response of tumours and their subpopulations, with implications for tumour recurrence. Characteristic features of the proneural subtype were a younger age at diagnosis and genomic alterations in platelet-derived growth factor receptor-α (*PDGFRA*) and tumour protein p53 (*TP53*), with high expression of genes associated with proliferation and neural and oligodendrocytic development [10, 17, 65]. Alternatively, the classical subtype was defined by chromosome 7 amplification paired with chromosome 10 loss, alterations in *EGFR* and an astrocytic and neural precursor gene expression signature [10, 17]. Mutations in the tumour suppressor genes neurofibromin (*NF1*) and phosphatase and tensin homolog (*PTEN*), as well as gene expression of mesenchymal and astrocyte markers chitinase 3-like 1 (*YKL40*), mesenchymal epithelial transition proto-oncogene receptor tyrosine kinase (*MET*) and cluster of differentiation 44 (*CD44*) are among defining features of the mesenchymal subclass [10, 65]. Although a neural subtype was initially defined, its existence as an independent subtype was later determined to result from contaminating non-tumour tissue [10, 29]. Differences in median survival time between glioblastoma, IDH-wildtype tumours assigned to mesenchymal, classical and proneural subgroups with limited intratumoural heterogeneity have been observed at 11.5, 14.7 and 17.0 months, respectively [29]. Further, a survival advantage for patients with classical and mesenchymal tumours in response to more intensive treatments (concurrent chemotherapy and radiation or > 4 cycles of chemotherapy) has been previously observed, with patients with proneural tumours not experiencing a difference in outcome between intensive and less intensive (non-current chemotherapy and radiotherapy or < 4 cycles of chemotherapy) therapy [10]. Strikingly, these transcriptional subtypes have been demonstrated to co-exist within the same tumour, showing spatial and temporal heterogeneity that confounds stratifications of treatment response and outcome [12, 13, 29]. Given that tumour subpopulations of diverse transcriptional subtypes will likely not respond identically to therapy, potential for residual surviving tumour cells to re-establish a tumour post-therapy is apparent.

**Fig. 1:**
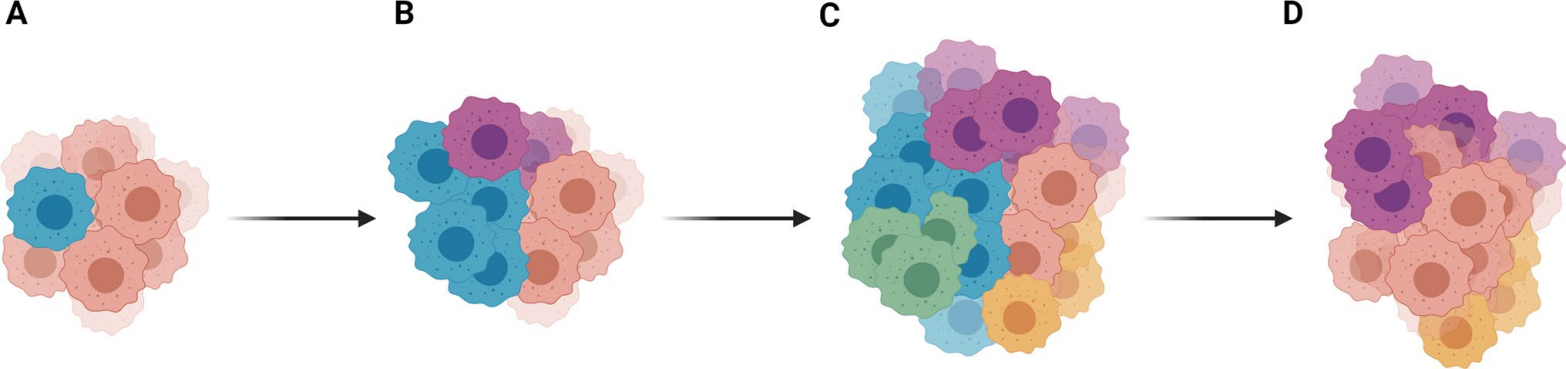
Intratumoural heterogeneity may arise by clonal evolution. **A**) A clonal population of neoplastic cells may acquire an additional mutation. **B**, **C**) Subsequent mutations may be acquired by these populations to generate progeny of increasing heterogeneity and abnormality. These populations may differentially expand. **D**) Resultant subpopulations may respond differently to selective pressures, such as therapeutic intervention, to alter subclone composition

The emergence of diverse subpopulations within a tumour is theorised to occur by processes of clonal evolution and/or the CSC hypothesis. The process of clonal evolution may generate heterogeneous cell populations within diffuse HGG tumours that vary in their susceptibility to therapeutic intervention (Fig. 1). In this model, a clonal population of neoplastic cells may variably acquire additional mutations to generate different subclones of increasing heterogeneity and abnormality [66–68]. These subclones may respond differently to selective pressure and nonuniformly expand under different conditions, affecting patient outcome [66–68]. Microarray gene expression analysis of different fragments from the same glioblastoma tumour and scRNA-seq data have demonstrated the existence of multiple transcriptional subtypes within a tumour despite a dominant proneural, classical or mesenchymal signature being assigned [12, 13]. Of particular clinical note, high intratumoural heterogeneity in proneural glioblastoma IDH-wildtype tumours was associated with a significant decrease in survival time [13]. Further, the presence of more than 10 subclones in paediatric HGG tumours was reported to be a significant predictor of shorter survival time [16]. These subclones may survive therapeutic insults and lead to the recurrence of a tumour with different properties to the primary, presenting challenges to treatment. Previous work demonstrated a change in transcriptional subtype associated with tumour recurrence in 63% of glioblastoma patients, with estimates of divergence time suggesting that recurrence clones existed early in tumour evolution [69]. Indeed, clones resistant to temozolomide chemotherapy have been isolated from treatment-naïve adult glioblastoma specimens [70]. Barcoding experiments of a non-small lung cancer cell line model have also suggested that clones responsible for relapse exist prior to treatment and are not a result of *de novo* mutations [71]. Moreover, analysis of the subclonal architecture of paediatric-type diffuse HGGs demonstrated the presence of multiple distinct subclonal lineages that experienced significant alterations in frequency throughout tumour evolution and in tumour recurrence, likely in response to changes in selective pressures [72]. The implementation of models that support such a diversity of tumour cell subtypes and allow for the study of tumour evolution and recurrence is therefore imperative to ascertain the effects of novel therapeutics.

The CSC hypothesis provides an alternate avenue for the generation of heterogeneous tumours. This model places CSCs capable of self-renewal and differentiation at the apex of hierarchically organised tumours, thereby generating phenotypically diverse progeny like normal stem cells [67, 73–75]. However, CSCs may also act as a unit of selection in clonal evolution, with GSCs harbouring diverse genetic alterations and tumorigenic potential reported within the same glioblastoma, IDH-wildtype tumours [67, 73–77]. Heterogeneous tumour populations in both IDH-wildtype and IDH-mutant gliomas have been demonstrated to resemble neurodevelopmental cellular hierarchies by scRNA-seq [15, 39, 78]. IDH-mutant astrocytomas and oligodendrogliomas have been reported to contain a highly proliferative neural precursor cell (NPC)-like population alongside less proliferative astrocyte-like and oligodendrocyte-like populations [15, 78]. Similarly, paediatric-type diffuse midline glioma, H3 K27-altered tumours were composed of a proliferating oligodendrocyte precursor cell (OPC)-like population and its astrocyte-like and oligodendrocyte-like progeny [39]. The implications of these hierarchies are apparent in the context of tumour grade, whereby astrocytoma, IDH-mutant tumours of higher malignancy contained higher proportions of cycling and undifferentiated cells [15]. Indeed, IDH-mutant gliomas harbour proliferative, NPC-like fractions of around 10%, while H3 K27-altered tumours with a significantly worse prognosis contain OPC-like compartments of ~ 80% [15, 39, 78]. Moreover, copy number variants in three high-grade astrocytoma, IDH-mutant tumours and two paediatric-type diffuse midline glioma, H3 K27-altered tumours identified variability in the fraction of cycling cells and in the pattern of astrocytic and oligodendroglial differentiation within different subclonal populations [15, 39]. This implies that different tumour subclones have different distributions of stem-like and differentiated compartments, establishing an interplay between cellular architecture and genetics.

Further scRNA-seq studies on glioblastoma, IDH-wildtype tumours revealed a cellular architecture containing OPC-like, NPC-like, astrocyte-like, and mesenchymal-like cell states in varying proportions across tumours [17]. These cellular states were favoured by particular genetic aberrations, with high levels of amplification of *EGFR*, *PDGFRA* and cyclin-dependent kinase 4 (*CDK4*) found in astrocyte-like, OPC-like and NPC-like states, respectively [17]. Accordingly, these genes are known regulators of astrocytes, OPCs and NPCs in normal neurodevelopment [17, 79–81]. Mutations in *NF1* favoured the mesenchymal-like cellular state, which was absent from IDH-mutant and H3 K27-altered gliomas [15, 17, 39, 78]. Extension of these findings to TCGA transcriptional subtypes determined that proneural tumours were enriched with OPC- and NPC-like cell states, classical tumours with the astrocyte-like cell state and mesenchymal tumours with the mesenchymal-like cell state, concurring with the frequencies of genetic aberrations [10, 17, 29]. Although genetic aberrations showed a bias for a particular state, tumour subclones contained cells in multiple cellular states, suggesting that genetic subclones are only partially responsible for intratumoural heterogeneity [17].

**Fig. 2:**
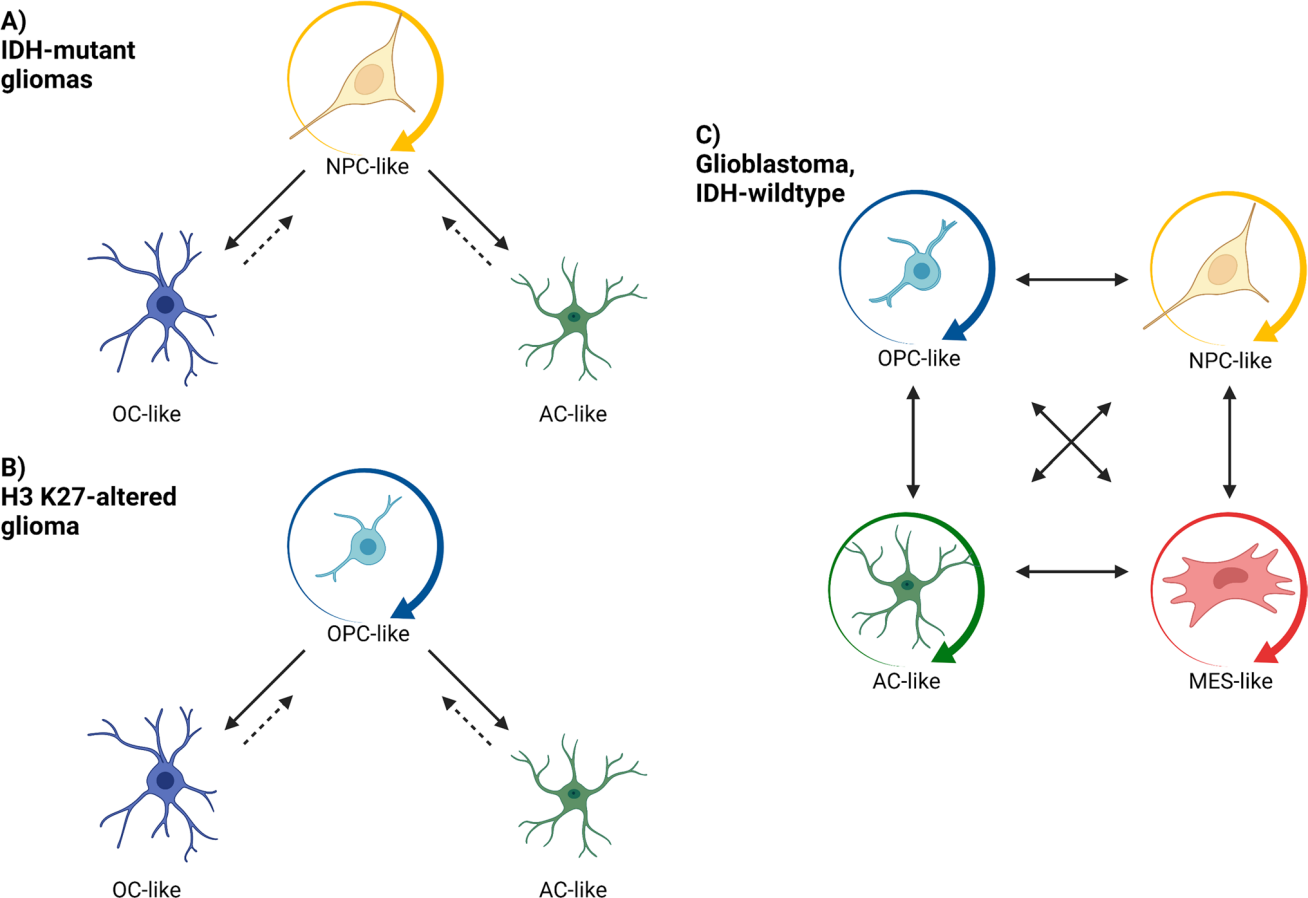
Cell states in high-grade gliomas are plastic. **A**) IDH-mutant astrocytomas and oligodendrogliomas contain a proliferative neural precursor cell (NPC)-like population capable of self-renewal and differentiating to give oligodendrocyte (OC)- and astrocyte (AC)-like progeny. Although less frequent, OC- and AC-like cells can dedifferentiate to regenerate NPC populations. **B**) Paediatric-type diffuse midline glioma, H3 K27-altered tumours contain an oligodendrocyte precursor cell (OPC)-like population with stemness features capable of generating differentiated OC- and AC-like progeny. It is expected that OC- and AC-like cells are capable of dedifferentiation to regenerate OPC-like populations. **C**) Glioblastoma, IDH-wildtype tumours contain OPC-, NPC-, AC- and mesenchymal (MES)-like compartments, each with stem-like and differentiated populations. Cells may move between cellular states through processes of differentiation and dedifferentiation

Plasticity between cellular states has been observed in HGG tumours. Barcoding experiments of patient-derived glioblastoma, IDH-wildtype orthotopic xenografts in immunocompromised mice produced glioblastoma cells that shared the same barcode but corresponded to different cellular states, indicating that one cell could generate progeny in other cellular states [17]. This suggests a plasticity between cellular states independent of genetics, supported by scRNA-seq findings of “hybrid” cells that appear to be between cellular states or TCGA transcriptional subtypes [13, 17]. Proliferation and stemness markers were found in some cells across all four cellular state compartments in glioblastoma, IDH-wildtype tumours, suggesting a heterogeneous cell population capable of acting as GSCs [17, 82]. Moreover, phylogenetic analysis of glioblastoma, IDH-wildtype tumours based on scRNA-seq data, DNA methylation profiles and copy number alterations (CNAs) determined that differentiation and dedifferentiation occurred to facilitate plasticity across OPC-, NPC-, astrocyte- and mesenchymal-like cell states [83]. Indeed, scRNA-seq studies have identified a stemness gradient in glioblastoma, IDH-wildtype tumours occupied continuously by cells expressing alternate levels of stemness genes, suggesting the presence of intermediate states between a stem-like and differentiated status [13]. Analysis of astrocytoma, IDH-mutant tumours revealed that although dedifferentiation occurred, rates were much lower than observed for glioblastoma, IDH-wildtype tumours and may thus explain the clearer cell hierarchy and more favourable outcome [83]. The cellular hierarchy in HGGs is therefore not strictly unidirectional and facilitates increased malignancy as well as the generation of new GSC populations (Fig. 2), which must be replicated in models in order to achieve clinically relevant outcomes.

The presence of cell populations of diverse differentiation status has been implicated in the resistance of HGG tumours to therapeutic intervention, in part due to the high adaptability and diversity of GSCs [22–24, 26]. GSCs have well established roles in tumour initiation, maintenance and regrowth following surgical resection and chemoradiotherapy [19–22, 24, 84]. Different stemness markers have been reported to mark distinct GSC populations that may arise from different cell types within tumours, with *CD133*, *CD24*, *CD44* and *NES* expression found to be enriched in OPC-, NPC-, mesenchymal- and astrocyte-like cell states, respectively [82, 85]. Further work highlights proneural and mesenchymal transcriptional subtypes of GSCs, with CD133^+^ proneural GSCs found to be enriched at the invasive tumour edge and CD109^+^ mesenchymal GSCs at the tumour core [25, 44–46]. Notably, clonal tracking of CD133^+^ proneural GSCs demonstrated an upregulation of *CD109* and *CD44* expression and an overall shift to the mesenchymal GSC phenotype in these cells in response to ionising radiation (IR), indicating the mesenchymal phenotype may be more resistant to IR insults as well as the capacity for an adaptability of the GSC population [25]. Indeed, GSCs have been demonstrated to utilise phenotypic shifts between proliferative and quiescent states to evade chemotherapy and regenerate tumours, given that chemotherapy primarily targets proliferative subpopulations. Treatment of endogenous murine glioma models with temozolomide eradicated proliferative cells, while quiescent GSCs were resistant and the source of proliferative and non-dividing tumour cells upon tumour recurrence [24]. Moreover, treatment of a GSC line harbouring a focal amplification of *PDGFRA* with PDGFR inhibitor dasatinib (1 µM) *in vitro* similarly demonstrated that although a large fraction of cells die upon treatment, a subset of proliferative cells transitioned to a Ki67^−^ quiescent state with innate treatment resistance that could re-establish a proliferative population upon dasatinib withdrawal [86]. Such rapid and reversible transitions between proliferative and quiescent GSC states have been demonstrated to occur on an epigenetic level through chromatin remodelling *via* H3K27me3 demethylation [86]. GSCs thus transition between diverse states with different therapeutic responses, highlighting treatment resistance and tumour recurrence as not solely properties of genetic heterogeneity, but also cellular potential. If preclinical models lack this innate diversity of cellular states found in HGG tumours, the results of novel therapeutic studies will likely be false positives when resistant phenotypes are absent.

The resistance of GSCs to therapeutic insults is not just a result of state transitions, but also an inherent property of a stemness phenotype. Both healthy and neoplastic stem cells display a superior DNA damage response (DDR) compared to their differentiated progeny, likely owing to higher proliferation rates and the requirement to generate and maintain tissues [87]. The DDR, encompassing DNA repair and cell cycle checkpoint pathways, plays an important role in the sensitivity of tumour cells to IR [22, 88, 89]. GSCs have been demonstrated to promote tumour radioresistance through preferential activation of the DDR. Bao et al. [22] showed that irradiation of adult and paediatric HGG cell lines and patient-derived specimens *in vitro* (2 Gy) and in orthotopic murine xenografts (5 Gy, 1–3 doses) increased the fraction of tumour cells positive for CD133 by 3-5-fold without inducing CD133 expression. This increase was associated with decreased caspase-3 activation and 4-5-fold lower annexin V staining in CD133^+^ cells compared to matched CD133^−^ cells in response to IR, suggesting higher fractions of CD133^+^ cells arise under these conditions due to resistance of a pre-existing GSC population to IR-induced apoptosis [22]. Indeed, significantly higher levels of activating phosphorylation of ataxia telangiectasia mutated (ATM), checkpoint kinase (CHK) 1, CHK2 and the ataxia telangiectasia and RAD3-related (ATR)-regulated checkpoint protein RAD17 were found in CD133^+^ cells in response to IR, compared to CD133^−^ cells [22]. Moreover, higher levels of baseline phosphorylation of RAD17 were found in CD133^+^ cells, suggesting this subpopulation may be primed to respond to DNA damage [22]. Indeed, higher expression of DNA double-strand break (DSB) repair protein RAD51 was found in GSCs marked by SOX2 and NES, with astrocytic differentiation of GSCs associated with a reduction in RAD51 expression [90]. Concordantly, immunoblotting of primary patient-derived glioblastoma cell lines from resected tumour specimens demonstrated elevated basal expression of DDR proteins CHK1, ATR and poly (ADP-ribose) polymerase 1 (PARP1) in the GSC fraction, as identified by CD133, SOX2, NES and oligodendrocyte transcription factor 2 (OLIG2) expression, compared to the bulk tumour fraction [91]. GSC populations were demonstrated to have a greater number of gamma histone H2A family member X (γH2AX) foci than matched differentiated cells in response to IR, further suggesting a higher ability of GSCs to initiate DSB repair [92]. Inhibition of ATM kinase (KU-55933, 10 µM, 1 h prior to irradiation) in GSCs and matched differentiated cells abrogated the higher survival and greater number of γH2AX foci evident in GSCs in response to IR [92]. Clinically, this observed radioresistance correlates with an increased proportion of CD133^+^ cells in HGG tumour recurrences following gamma knife surgery and external beam radiation, compared to the primary tumour (16.5 ± 12.12.2% vs 1.23 ± 2.36%, *n* = 10) [93]. Thus, GSCs may survive radiotherapy and promote tumour recurrence through mechanisms that include the DDR.

The heterogeneity both within and between tumours discussed above and the effects on patient prognosis and therapeutic efficacy have significant implications for glioma tumour modelling. In an effort to develop clinically relevant treatment strategies, preclinical models must therefore be conducive to the maintenance of GSC and other diverse cellular subpopulations within glioma tumours. Moreover, they must support the diverse underlying aetiologies of tumour types and recapitulate the evolution of these tumours. Two-dimensional (2D) cell culture models are perhaps the most widely established preclinical platform for the interrogation of novel therapeutics. However, the culture of initially highly heterogeneous primary glioma cell lines under serum-containing monolayer culture conditions for one month led to the population becoming homogenous [94]. With the accuracy of preclinical drug screening dependent on tumour heterogeneity, as detailed above, the design of more relevant models must consider what drives and supports this tumour diversity if we are to improve the success rate of oncology clinical trials. Recent evidence suggests that such intratumoural heterogeneity is not random, but a consequence of the complex HGG microenvironment [95–97].

### The microenvironment shapes tumour heterogeneity, progression and therapy resistance

Stronger associations of specific cellular states and gene expression signatures with regional and structural features support the notion that microenvironmental interactions favour particular states [25, 44–46]. Analysis of neoplastic cells from glioblastoma, IDH-wildtype tumours at the infiltrating edge by scRNA-seq determined a common gene signature, regardless of patient origin, that most closely resembled OPCs [97]. Infiltrating tumour cells showed upregulation of genes involved in cell-cell adhesion, size regulation, the inhibition of apoptosis, energy production and CNS development [97]. Alternatively, neoplastic cells at the tumour core displayed an enrichment of genes related to hypoxia, including *HIF1A*, vascular endothelial growth factor A (*VEGFA*) and carbonic anhydrase 9 (*CA9*) [97]. Moreover, transcriptional profiling of distinct structural regions of glioblastoma taken by laser capture microdissection identified strong enrichment of the hypoxia-related mesenchymal subtype in regions of necrosis at the tumour core in 37 patient-derived tumours, concurring with the association of mesenchymal GSCs with the hypoxic tumour core [25, 95, 96]. Proneural expression signatures were found in bulk tumour samples and GSCs at the outer tumour boundary in contact with neural tissue and brain vasculature, while intermediate regions contained a mixture of classical and proneural signatures [95, 96]. However, cells expressing mesenchymal genes have been reported at the infiltrating tumour edge and cells with proneural signatures have been found in the tumour core [45]. Nonetheless, the strong association of these transcriptional subtypes with particular regions suggests that cell-cell interactions and nutrient availability support specific phenotypes. As such, three main glioma tumour microenvironmental niches have been described, namely the perivascular, hypoxic and invasive niches that provide specific cues promoting the growth, maintenance and protection of distinct subsets of tumour cells [26, 28, 30] (Table 2). Predominant cell types within the glioma microenvironment include neurons, astrocytes, vascular cells, immune cells, NPCs and oligodendrocytes, as well as neighbouring neoplastic cells. With an established ability of exogenous factors to influence the phenotype of a tumour cell (Table 2), understanding the pathobiology of HGG cells in the context of their microenvironment is critical in the development of clinically relevant therapies.

**Table 2:** Microenvironmental cues influence HGG cell phenotypes and therapeutic response

Signalling molecule/ condition from microenvironment	Cell type of expression/ release	Interacting/ responsive molecule(s) of glioma cell	Effect on tumour	References
CXCL12	Endothelial cells, astrocytes and neurons	CXCR4	Promotes invasive phenotype, inhibits apoptosis and promotes proliferation	[98–100]
Bradykinin	Endothelial cells	B2R	Promotes motility of glioma cells towards blood vessels and deeper invasion of brain tissue	[101, 102]
Jagged and Delta-like ligands	Endothelial cells	NOTCH receptor	Maintains the GSC fraction by promoting GSC self-renewal, proliferation and expression of stemness-related genes. Promotes radioresistance	[103–107]
Nitric oxide	Endothelial cells	sGC	Promotes NOTCH signalling	[104, 107]
SHH	Endothelial cells	PTCH1 and PTCH2	Increases expression of genes involved in proliferation, stemness, angiogenesis and survival	[108]
Interleukin-8	Endothelial cells	CXCR2	Increases invasion, expression of stemness-related genes and tumour growth	[109]
CD9	Endothelial cells	Interleukin-6 receptor gp130	Increases expression of stemness-related genes and the proliferation of GSCs	[110]
Long-term hypoxia	N/A	HIF2A	Promotes the expression of stemness-related genes and favours the survival of the mesenchymal GSC phenotype	[95]
Acute hypoxia	N/A	HIF1A	Induces a metabolic shift towards glycolysis and promotes quiescence	[111, 112]
Acute hypoxia	N/A	CD133, CD44	Increases stemness properties	[113]
Acute hypoxia	N/A	HIF1A, BIRC3, FTL	Increases the resistance of HGG cells to radiotherapy and chemotherapy through mechanisms that include the inhibition of apoptosis	[114, 115]
Cycling hypoxia	N/A	HIF1A, GOT1, GSH	Reduces levels of intracellular reactive oxygen species to increase radioresistance	[116–118]
Cycling hypoxia	N/A	HIF1A, ABCB1 drug efflux pump	Increases resistance to chemotherapy by reducing intracellular accumulation of chemotherapeutics	[119–121]
Hypoxia	N/A	HIF1A, VEGF	Induces angiogenesis	[122, 123]
Acute hypoxia	N/A	HIF1A, CXCR4, CD133	Promotes the expansion of CD133^+^ and CXCR4^+^ GSCs, inhibits differentiation and promotes migration	[98, 122, 124]
Acute and chronic hypoxia	N/A	HIF1A, HIF2A, NOTCH pathway, VEGF signalling	Promotes transdifferentiation of GSCs into endothelial cells	[125–128]
JAG1	Neurons	NOTCH1	Supports GSC maintenance, survival and migration	[129, 130]
NLGN3	Neurons	FAK, PI3K-mTOR, SRC kinase and RAS signalling cascades	Promotes tumour cell proliferation. Increases expression of synapse-related genes and the formation of neuron-to-glioma synapses	[32, 131, 132]
Glutamate	Neurons	AMPARs	Induces glioma cell proliferation and increased migration speed	[132, 133]
CCL5	TAMs	CaMKII, MMP2	Promotes an invasive glioma cell phenotype	[134–136]
MMP14	TAMs	pro-MMP2	Supports invasion of glioma cells	[135, 136]
Pleiotrophin	TAMs	PTPRZ1	Increases tumour growth and the SOX2^+^ GSC fraction	[33]
TGFB1	TAMs	TGFBR2, MMP9	Increases the invasive ability of CD133^+^ GSCs	[137]

ABCB1, adenosine triphosphate-binding cassette subfamily B member 1. AMPAR, α-amino-3-hydroxy-5-methyl-4-isoxazole propionic acid receptor. B2R, bradykinin 2 receptor. BIRC3, baculoviral inhibitor of apoptosis repeat-containing protein 3. CaMKII, Ca^2+^/calmodulin-dependant protein kinase II. CCL5, C-C motif chemokine ligand 5. CD9, tetraspanin CD9. CD44, cluster of differentiation 44. CD133, cluster of differentiation 133 (prominin-1). CD44, cluster of differentiation 44. CXCL12, C-X-C motif chemokine ligand 12. CXCR2, C-X-C motif chemokine receptor 2. CXCR4, C-X-C motif chemokine receptor 4. FAK, focal adhesion kinase. FTL, ferritin light chain. GOT1, glutamic-oxaloacetic transaminase 1. gp130, glycoprotein 130. GSC, glioma stem cell. GSH, glutathione. HGG, high-grade glioma. HIF1A, hypoxia-inducible factor 1α. HIF2A, hypoxia-inducible factor 2α. JAG1, Jagged 1. MMP2, matrix metalloproteinase 2. MMP9, matrix metalloproteinase 9. MMP14, matrix matalloproteinase 14. NLGN3, neuroligin-3. NOTCH1, Notch receptor 1. PI3K-mTOR, phosphatidylinositol-3-kinase-mammalian target of rapamycin. PTCH1, patched 1. PTCH2, patched 2. PTPRZ1, protein tyrosine phosphate receptor type Z1. sGC, soluble guanylate cyclase. SHH, sonic hedgehog. SOX2, sex determining region Y-box 2. TAM, tumour-associated macrophage. TGFB1, transforming growth factor beta 1. TGFBR2, transforming growth factor beta receptor 2. VEGF, vascular endothelial growth factor

HGGs are highly vascularised tumours that display strong capacity for angiogenesis and vessel co-option in the densely vascularised brain microenvironment. Estimates suggest that as few as 3 glioma cells are required to occupy the space between adjacent brain microvessels, highlighting their prominence in this environment [138]. Interactions between HGG cells and neighbouring endothelial cells in the perivascular niche have been demonstrated to influence cell phenotype and support tumour diversity and invasion. Indeed, U251 human glioma cells demonstrated preferential migration along blood vessels in both grey and white matter during organotypic murine brain slice culture, while 85% of human glioma cells were shown to migrate towards vasculature when injected into the murine brain [101, 139]. Cell-cell signalling pathways that contribute to the localisation of glioma cells around blood vessels and their invasive, therapy resistant phenotype include C-X-C motif chemokine ligand 12 (CXCL12) to C-X-C motif chemokine receptor 4 (CXCR4), and bradykinin to bradykinin 2 receptor (B2R) [98–102] (Table 2). A significant increase in glioma cell line motility towards an increasing bradykinin gradient has been observed *in vitro*, and could be prevented by the presence of bradykinin 2 receptor (B2R) antagonists (HOE 140 or Bradyzide) [101]. Moreover, human HGG cell lines showed reduced association with blood vessels in rat brain slices in the presence of HOE 140 (5 µM) and following shRNA-mediated B2R knockdown, whereas the addition of exogenous bradykinin (1 µM) resulted in deeper invasion of brain tissue [101]. Immunocytochemistry of glioma patient tissue revealed higher levels of B2R found with higher grades of malignancy and within perivascular regions, further suggesting this signalling pathway impacts tumour dynamics [101]. Orthotopic murine transplantation of human U87MG glioma cells has also demonstrated a CXCR4-dependent migration towards CXCL12-expressing blood vessels in the subventricular zone (SVZ) that could be prevented by AMD3100 (1.25 mg/kg) and by CXCR4 knockdown [140]. Moreover, mice containing orthotopic glioma xenografts undergoing radiation treatment showed increased overall survival time when CXCR4 was knocked down in glioma cells [99]. This was associated with increased caspase-3 activation compared to CXCR4 wildtype glioma cells, suggesting that CXCR4 promotes radiation resistance in cells in the perivascular niche by the inhibition of apoptosis [99]. Indeed, CXCR4 antagonism (PRX177561, 1 µM) has been demonstrated to increase apoptosis, reduce proliferation, promote GSC differentiation and inhibit CXCL12-dependent migration *in vitro* [100]. With vasculature interactions associated with invasive and therapy-resistant glioma cell populations, an inherent requirement for vasculature to be present in relevant glioma models is apparent.

**Fig. 3:**
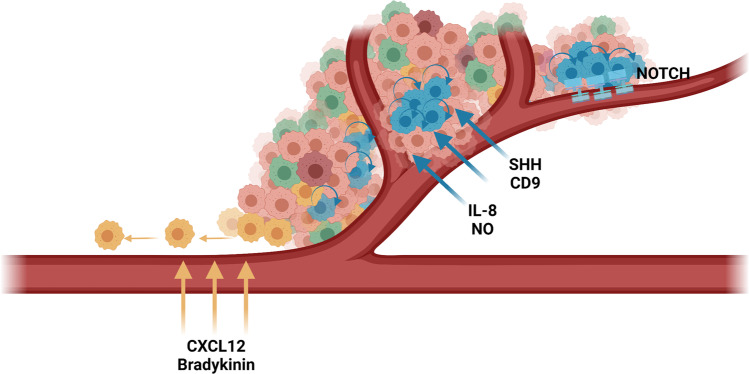
The perivascular niche promotes glioma cell migration and supports the therapy resistant glioma stem cell (GSC) phenotype. Endothelial cells in the perivascular niche release chemoattractants C-X-C motif chemokine ligand 12 (CXCL12) and bradykinin to increase the migration and invasive ability of glioma cell subpopulations expressing C-X-C motif chemokine receptor 4 (CXCR4) and bradykinin 2 receptor (B2R), respectively. These invasive populations migrate along pre-existing vasculature to invade healthy brain tissue. Endothelial cells also release sonic hedgehog (SHH), tetraspanin CD9 (CD9), interlukin-8 (IL-8) and nitric oxide (NO), promoting the stemness, self-renewal and survival of glioma stem cell (GSC) subpopulations. NOTCH receptors expressed on GSC surfaces bind NOTCH ligands, including delta-like 4 (DLL4), expressed on endothelial cells to facilitate the maintenance of the GSC state

Cell-cell signalling between endothelial and glioma cells has also been demonstrated to support the presence GSC subpopulations by promoting stemness, self-renewal and survival pathways. Neural stem and progenitor cells in the adult CNS are restricted to the subventricular zone of the lateral ventricles and the subgranular zone of the hippocampal dentate gyrus, typically in association with local dense vasculature that provides maintenance cues [141–145]. Likewise, the perivascular niche provides cues for GSCs to self-renew and proliferate through established developmental pathways, including NOTCH and sonic hedgehog (SHH) signalling [31, 103, 106, 146]. Notably, the GSC fraction of neurosphere cultures was depleted in the absence of NOTCH signalling, with mRNA expression of GSC markers *CD133*, *NES*, *OLIG2* and *BMI1*, as well as the fraction of cells positive for CD133 protein, reduced by NOTCH pathway inhibitor γ-secretase inhibitor 18 (GSI-18) in a dose-dependent manner [103]. Further, shRNA-mediated knockdown of NOTCH-ligands delta-like ligand 4 (DLL4) or Jagged 1 (JAG1) in human brain microvascular endothelial cells prior to intracranial co-implantation with human HGG neurospheres significantly reduced xenograft size and the CD133^+^ GSC fraction, suggesting endothelial cells support tumour growth and GSC maintenance [106]. Additional mechanisms that support the maintenance of GSC populations within the perivascular niche include nitric oxide, interleukin-8 and CD9 signalling, with siRNA-mediated knockdown of any of these endothelial cell-derived molecules or their HGG-associated receptor culminating in decreased expression of stemness-related genes and reduced proliferation of GSCs [104, 107–110] (Table 2). Taken together, these findings indicate that the perivascular niche provides selective cues that support the maintenance, malignancy and therapeutic resistance of particular cell states (Fig. 3). With the established therapeutic resistance of GSCs to chemoradiotherapy, interactions in the perivascular niche that promote this robust phenotype are critical considerations in HGG modelling and therapeutic development, as true effects of therapeutics cannot be realised in the absence of the resistant cell phenotypes they support.

HGG cells experience oxygen gradients and conditions of hypoxia *in vivo* that also result in intratumoural heterogeneity. Hypoxia has an established role in the maintenance of an aggressive, therapy resistant GSC phenotype with strong connection to the mesenchymal subtype [10, 13]. HIFs regulate cellular response to hypoxic stress, with HIF2A predominantly activated during long-term hypoxia and known to induce the expression of pluripotency associated genes *OCT4*, *SOX2*, and *NANOG* [147–150]. As such, immunohistochemistry experiments on patient-derived HGG tissue demonstrated the expression of HIF2A predominantly in regions of necrosis, with this expression colocalised with GSC marker CD133 [151]. Modulation of cell phenotype by microenvironment is apparent during conditions of hypoxia, with expression of *HIF2A*, *OCT4*, *NANOG* and *c-MYC* upregulated in non-stem HGG cells *in vitro* during hypoxic stress (1% O_2_, 10 days), as determined by RT-PCR [152]. Moreover, ectopic expression of HIF2A (72 h) promoted the upregulation of *OCT4*, *NANOG* and *c-MYC* transcripts and thus the induction of a GSC phenotype in non-stem cell populations derived from adult and paediatric HGG patient biopsy lines *in vitro*, as well as the formation of significantly larger tumours in immunocompromised mice [152]. While hypoxia may induce the proliferation of some GSC subtypes, the induction of quiescence may occur in others in response to hypoxic stress and favours the mesenchymal subtype [95, 122, 153–155]. Analysis of NPCs and proneural and mesenchymal GSCs under hypoxic stress demonstrated preferential survival of mesenchymal GSCs through upregulation of mesenchymal markers YKL40 and CD44 and sustained BMI1 expression, whereas most NPCs and proneural GSCs lost BMI1 expression and viability [95]. Thus, an active selection process occurs at the hypoxic tumour core that favours the presence of an aggressive mesenchymal GSC phenotype, concurring with previous associations of the mesenchymal phenotype with necrotic tumour regions [25, 95, 96]. Hypoxia response genes have also been inversely correlated with cell cycle genes in scRNA-seq analysis of glioblastoma, IDH-wildtype tumours, indicating a pause in cell proliferation in conditions of hypoxia [13]. Accordingly, HIF1A^+^ GSCs with a phosphorylated serine 2 residue of RNA polymerase II, a marker of quiescence, were found in perinecrotic regions of HGG patient tissue [112]. Hypoxia is thus an important microenvironmental factor in the maintenance of particular stem cell populations.

Hypoxia induces a variety of intracellular signalling pathways that promote therapy resistance (Table 2). Hypoxia (1% O_2_, 24 h) has been shown to induce the expression of anti-apoptotic protein baculoviral inhibitor of apoptosis repeat-containing protein 3 (BIRC3), expressed highly in the TCGA mesenchymal glioblastoma subtype, through HIF1A in *in vitro* HGG models [114]. Importantly, BIRC3 knockdown by siRNA impaired the resistance of HGG cells to radiotherapy (2–8 Gy) observed in hypoxia, implicating this anti-apoptotic protein in hypoxia-mediated increases in HGG cell survival [114]. Moreover, increases in BIRC3 expression have been demonstrated to occur secondary to the acquisition of chemoradiotherapy resistance by HGG cells *in vitro*, as well as in HGG recurrence through patient biopsies, and associated with a worse prognosis [156]. Hypoxia may thus promote the survival of a distinct subset of cells that may repopulate a tumour to cause relapse. Further evidence for hypoxic microenvironmental influence in chemotherapy and radiotherapy resistance surrounds expression of the adenosine triphosphate (ATP)-binding cassette sub-family B member 1 (ABCB1) drug efflux pump, as well as reduced reactive oxygen species (ROS) levels. While tumour hypoxia may be chronic in core regions, conditions of cycling hypoxia are also apparent in tumour regions with intermittent or poor blood supply by new, often abnormal blood vessels and can induce expression of HIF1A [120]. Cycling hypoxia has been demonstrated to induce the upregulation of aspartate-aminotransferase glutamic-oxaloacetic transaminase 1 (GOT1) protein in an adult HGG cell line, causing increased levels of antioxidant glutathione (GSH) and reduced intracellular ROS levels [118]. This was associated with increased radioresistance, given that IR can induce apoptosis through the generation of ROS by water radiolysis [116–118]. Cycling hypoxia also has been demonstrated to increase *ABCB1* expression and enhance its function of reducing intracellular accumulation of xenobiotic compounds, including chemotherapeutics [119–121]. Indeed, resistance to chemotherapeutics doxorubicin and BCNU was enhanced by pretreatment of HGG cell lines with cycling hypoxia (3 cycles of 0.5–1% O_2_ for 1 h interrupted by 5% CO_2_ and air for 30 min) [120]. Knockdown of *ABCB1* by siRNA decreased observed resistance following cycling hypoxia treatment [120]. Moreover, expression of *ABCB1* with *HIF1A* in murine orthotopic xenografts was localised to areas of tumour cycling hypoxia [120]. Thus, the importance of niche microenvironments in maintaining resistant subpopulations is highlighted.

While many other highly malignant solid cancers rely on lymphatic and vascular invasion to spread, HGGs adeptly migrate along white matter tracts and blood vessels to form satellite tumours [129, 139, 157–159]. Thus, these regions at the tumour periphery are major features of the invasive niche. In a similar manner to brain vasculature, HGG cells may migrate along white matter tracts by CXCL12-CXCR4 signalling, with high expression of CXCL12 by neurons and CXCR4 by associated glioma cells [98]. In a similar manner to blood vessels, a preferential localisation of CD133^+^ GSCs to white matter tracts has been described, suggesting further GSC maintenance mechanisms occur in these regions of the invasive front [129]. Indeed, adult HGG patient tissues showed that NOTCH1 was expressed in these GSCs and JAG1 was found in white matter tracts at the invasive front [129]. Moreover, co-culture with neurons expressing Tau1 and JAG1 revealed higher neuronal axon tropism of CD133^+^ GSCs than CD133^−^ glioma cells that could be reduced by shRNA-mediated *NOTCH1* knockdown, further suggesting features of the brain microenvironment support specific cell phenotypes [129].

The formation of tumour microtubes (TMs) in astrocytomas is another critical feature of the invasive niche and is associated with stemness. Transplantation of glioblastoma, IDH-wildtype GSC lines into mouse brains revealed the formation of long cellular protrusions at the invasive front that interconnected single tumour cells and increased in number with tumour progression [160]. These TMs formed between cells during cell division as well as between unrelated cells and transported nuclei, microvesicles and mitochondria [160]. The connectivity of TMs was mediated by gap junction-forming protein connexin 43 (Cx43) and enabled the propagation of intercellular calcium waves, with shRNA-knockdown of *Cx43* significantly reducing the synchronicity of calcium waves and tumour size in mice [160]. Importantly, TM-connected glioma cells showed greater resistance to radiotherapy (3 × 7 Gy) mediated by an ability to distribute otherwise lethal increases in intracellular calcium levels, which was reduced by Cx43 knockdown *in vivo* [160, 161]. Moreover, GSCs connected to at least 2 other tumour cells by TMs showed a significantly higher proportion of surviving cells following temozolomide treatment (100 mg/kg, 3 days), while unconnected tumour cells showed significantly higher levels of cell death [162]. The occurrence of TMs *in vivo* only implies that cell-cell interactions within the brain microenvironment influence TM formation and behaviour [162]. Indeed, TMs have been demonstrated to be a primary site of contact between glioma cells and neurons, forming neurogliomal synapses [133]. The absence of TMs from traditional cell culture platforms is thus an important factor to consider when modelling HGGs and the effects of novel therapeutics, given that TMs play a critical role in therapy resistance.

Signalling from neurons in the surrounding microenvironment has also been demonstrated to drive adult and paediatric HGG tumour progression [132, 133]. Higher deep cortical projection neuron activity during optogenetic stimulation of the mouse prefrontal cortex has been demonstrated to increase the proliferation of patient-derived orthotopic xenografts of paediatric cortical HGG, as assessed by EdU incorporation and Ki67 immunostaining [32]. Increases in proliferation were most pronounced following the release of synaptic adhesion molecule neuroligin-3 (NLGN3) from microenvironmental neurons [32]. NLGN3 is important for synaptic function and plasticity, experiencing activity-regulated shedding from healthy neurons and OPCs into the surrounding microenvironment by the action of the a-disintegrin and metalloproteinase domain-containing protein 10 (ADAM10) protease [32, 131, 163, 164]. Patient-derived orthotopic xenografts of adult and paediatric HGGs experienced significant growth inhibition in *Nlgn3* knockout mice, indicating a substantial role of microenvironmental Nlgn3 in HGG progression [131]. Similarly, ADAM10 inhibition (GI254023X, 200 nM or INCB7839, 50 mg kg^−1^) in mice afflicted with paediatric HGG orthotopic xenografts resulted in significant growth reduction relative to vehicle controls, as seen in *in vitro* models of adult HGG [131, 165]. These findings thus suggest a strong dependence of HGG proliferation on neuronal activity and paracrine signalling in the surrounding brain microenvironment, predominantly through NLGN3 release. Translating NLGN3 findings from preclinical models to patient populations determined that lower levels of global and peritumour oscillatory brain activity, measured macroscopically by magnetoencephalography, indeed corresponded to lower levels of NLGN3 and longer progression-free survival in patients aged greater than 17 years with WHO grade 2, 3 or 4 diffuse glioma [166, 167]. Strikingly, increased expression of NLGN3 has been correlated with HGG recurrence in deep brain regions, while greater expression of ADAM10 has been associated with a higher tumour grade in human glioma surgical specimens, supporting a critical role of these molecules in glioma malignancy [165, 168].

In addition to paracrine signalling pathways, neuron-to-glioma cell synaptic communication has also been demonstrated to drive adult and paediatric tumour progression [132, 133]. Electron microscopy of resected adult primary glioblastoma tissue and of paediatric and adult HGG patient-derived xenograft murine models provided structural evidence for the formation of synapses between presynaptic neurons and postsynaptic glioma cells [132, 133]. Chemically, confocal microscopy detailed a punctate pattern of α-amino-3-hydroxy-5-methyl-4-isoxazole propionic acid receptors (AMPAR) on HGG cells overlapping with neuronal glutamatergic presynaptic vesicle clusters to concur with an observed NLGN3-induced increase in glutamate receptor subunit genes [131, 133]. The functionality of neuron-to-glioma synapses has subsequently been demonstrated by electrophysiological patch clamp recordings showing depolarising inward fast excitatory postsynaptic currents (EPSCs) in subsets of cells from resected glioblastoma tissue and HGG xenograft models [132, 133]. These EPSCs were strongly inhibited by AMPAR antagonists NBQX (10 µM) and CNQX (10 µM), reduced by calcium-permeable AMPAR selective antagonist NASPM (50 µM) and blocked by voltage-gated sodium channel antagonist tetrodotoxin (0.5–1 µM), indicating that presynaptic neuronal action potentials induce EPSCs in postsynaptic glioma cells through glutamate-AMPAR-mediated signalling in a manner reminiscent of neuron-to-OPC synapses [132, 133, 169]. HGG cells may thus integrate into the electrical circuitry of the surrounding brain microenvironment to facilitate tumour progression.

Slow inward currents (SICs) consistent with those observed in astrocytes were also found in subsets of glioma cells and were predominantly evoked by extracellular potassium accumulation from neuronal activity [132, 133]. Accordingly, these currents were inhibited by tetrodotoxin (0.5–1 µM) and largely reduced by barium ion solution (200 µM), which blocks inward potassium channels [132, 133]. As in astrocytes, calcium transients induced by potassium flux were also demonstrated to propagate across the glioma network through gap junctions and were inhibited by gap junction blockers carbenoxolone (100 µM) and meclofenamate (100–200 µM) [132, 133]. Gap junction coupling in adult and paediatric gliomas has been demonstrated to occur through TMs, with strong TM connectivity an established driver of incurable gliomas and a major site of AMPAR-containing synaptic contacts [132, 133, 160, 162]. Depolarisation of paediatric HGG xenografts in the murine cortex by optogenetic stimulation significantly increased the percentage of proliferating Ki67^+^ cells, while mice treated with AMPAR-blocking drug perampanel (0.75 mg kg^−1^, 5 days per week for 3 weeks) or with meclofenamate sodium (20 mg kg^−1^, 5 days per week for 2 weeks) experienced a 50% reduction in paediatric HGG xenograft proliferation compared to vehicle controls [132]. Moreover, these electrical properties of HGG tumours also displayed heterogeneity, with ~ 5–10% of paediatric HGG cells exhibiting EPSCs and ~ 40% SICs [132]. These proportions were influenced by cellular state properties of the tumour, being OPC-like and astrocyte-like compartments for EPSCs and SICs, respectively [132]. Together, these results suggest that HGGs integrate into the electrical circuitry of the surrounding brain microenvironment, experiencing neuronal activity-dependent depolarisations that induce proliferative and invasive pathways to promote tumour progression in a manner dependent on cell state composition. Replicating these neuron-to-glioma cell interactions in a relevant microenvironment is therefore essential to appropriately model tumour cell behaviour and response to therapy.

Tumour-associated macrophages (TAMs), comprising brain-resident microglia and infiltrating macrophages, constitute ~ 40% of the tumour cell mass of adult HGGs and form an additional microenvironmental consideration in the effective modelling of these tumours [135]. Although similar quantitative assessments of TAMs in paediatric HGGs are lacking, their presence in paediatric tumours has been described [170, 171]. Given that they form a considerable proportion of the tumour mass, TAMs are highly relevant to HGG modelling and pathobiology. While macrophages from blood circulation are absent from the brain during normal physiological conditions, breakdown of the blood-brain barrier during HGG progression facilitates the infiltration of circulating macrophages into the tumour mass where they are almost indistinguishable from resident microglia [135, 172]. Recruitment of TAMs to the tumour mass may occur by a number of pathways, including the secretion of CXCL12, fractalkine, glial-cell derived neurotrophic factor (GDNF), monocyte chemoattractant protein-1 (MCP-1) and macrophage colony-stimulating factor 1 (CSF1) from glioma tumours [135]. TAMs are a heterogeneous population that have tumour-suppressive (M1) or tumour-supportive (M2) properties, thus influencing the glioma landscape [30]. Notably, TAMs promote an invasive glioma phenotype, with *in vitro* studies indicating that co-culture of murine glioma cells with microglia increased migration to levels higher than co-culture with oligodendrocytes and endothelial cells, or glioma cells alone [173]. Mechanistically, TAM-induced invasive properties of glioma cells converge on MMPs, which have well established roles in ECM remodelling and tumour invasion [174]. Expression of chemokine C-C ligand 5 (CCL5) by TAMs resulted in increased intracellular Ca^2+^ in glioma cells and subsequent phosphorylation of Akt and Ca^2+^/calmodulin-dependent protein kinase II (CaMKII), increasing the expression of cleaved MMP2 that could be prevented by siRNA-mediated CaMKII knockdown [134]. Moreover, culture of glioma cells with activated TAM-conditioned media induced *MMP2* mRNA expression, suggesting a direct influence of TAMs on glioma gene expression [134]. However, MMP2 is initially released in a pro-MMP2 form that needs to be cleaved for activation by proteases including MMP14, expressed in the membranes of activated TAMs [135, 136]. MMP14-expressing TAMs were closely associated with glioma cells at the invasive edge in a murine glioma model to further suggest their supportive role in glioma invasion [136]. TAMs thus favour an invasive glioma cell phenotype that supports tumour progression.

Recruitment of TAMs to HGG tumours appears to be favoured by particular cell states. Gene expression analysis of human glioblastoma, IDH-wildtype tumours determined a stronger association of TAMs with the mesenchymal subtype influenced by characteristic *NF1* aberrations, with shRNA-mediated *NF1* knockdown reducing the recruitment of microglia to glioma cells in *in vitro* studies [29]. Furthermore, comparisons between primary and recurrent glioblastoma, IDH-wildtype tumours demonstrated that proneural and classical tumours that transitioned to a more resistant mesenchymal subtype at recurrence showed higher infiltration of TAMs at recurrence [29]. Short-term relapse tumours showed higher levels of M2 macrophages than long-term relapse tumours after radiotherapy, suggesting M2 macrophages may play a role in radiation resistance [29]. The recruitment of TAMs has also been associated with a poorer prognosis in paediatric HGGs to further suggest their critical role [171]. Accordingly, co-implantation of bone marrow-derived macrophages with melanoma cells into mice increased radioresistance in a manner dependent on tumour necrosis factor α (TNFα) signalling, while macrophage depletion by liposomal clodronate enhanced the antitumour effects of radiation (20 Gy) to suggest TAMs have malignancy-promoting properties across cancers [175]. Specific interactions with GSCs have also been described, with the density of TAMs correlating with the presence of GSCs at perivascular, hypoxic, and invasive niches [137, 176, 177]. Periostin (POSTN), expressed highly and secreted by GSCs but not non-stem tumour cells, correlated with TAM density in HGG patient tumours and promoted TAM recruitment [178]. Inhibition of TAM recruitment by shRNA-mediated knockdown of *POSTN* in GSCs was associated with reduced tumour growth and extended survival in mice bearing orthotopic xenografts, suggesting TAMs support GSC proliferation [178]. Indeed, M2 TAMs have been demonstrated to secrete pleiotrophin, with GSCs preferentially expressing its receptor, receptor-type tyrosine-protein phosphatase zeta (PTPRZ1) [33]. Silencing of *PTPRZ1* in GSCs by shRNA-mediated knockdown inhibited the growth of GSC-derived xenografts in mice, reduced the fraction of tumour cells expressing GSC marker SOX2 and extended the survival of mice bearing xenografts [33]. Moreover, TAMs at the invasive front produced high levels of transforming growth factor- β1 (TGFB1), which enhanced the invasive ability of CD133^+^ GSCs by inducing the expression of MMP9 in GSCs [137]. Knockdown of type II TGFB receptor (TGFBR2) by shRNA reduced the invasion of implanted murine glioma cell line-derived tumours into surrounding murine brain tissue, with the presence of CD133^+^ cells at the invasive front markedly reduced [137]. TAMs thus promote tumour progression and resistance, with specific effects on GSC proliferation, maintenance, and invasion.

## Models that accurately recapitulate the range and plasticity of cellular states present in HGGs are critical to the development of relevant therapies

**Fig. 4:**
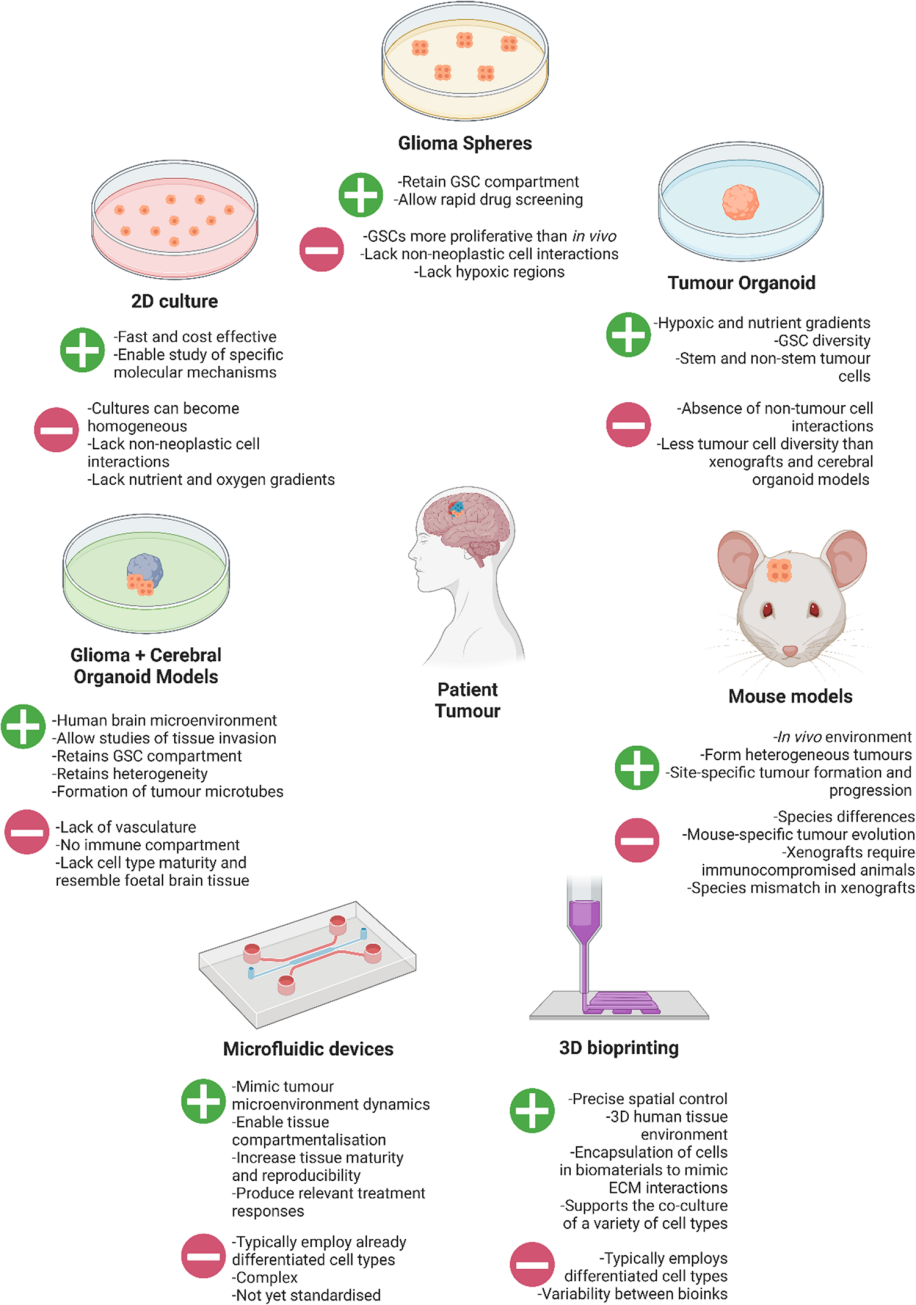
Advantages and disadvantages of modelling platforms. Diffuse high-grade gliomas have been modelled through two-dimensional (2D) cell culture, culture as glioma spheres under stem-promoting conditions, three-dimensional (3D) organoid culture of glioma cells, the generation of mouse models, by the co-culture of glioma cells and cerebral organoids, by microfluidic devices and by 3D bioprinting. Each have inherent advantages and limitations that require consideration in research and development. GSC, glioma stem cell. ECM, extracellular matrix

Evidently, HGG tumours have a high degree of heterogeneity that is heavily influenced by the surrounding microenvironment. With diverse cell states known to differentially respond to therapeutic intervention as well as receive microenvironmental cues, assessing the impact of novel treatments requires an appropriate context that recapitulates critical HGG features. Current animal and 2D cell culture models are suboptimal for the study of HGGs and may partially explain the lack of substantial improvements in outcome over the last three decades [4, 6–8, 179, 180]. This is perhaps most strongly reflected by the failure of 95% of preclinical anticancer agents to be approved following clinical trials [181]. Although reasons for this high drug attrition rate are multi-faceted, the inability of 2D models to recapitulate tumour heterogeneity and the 3D microenvironment together with the disparities between human and animal physiology strongly contribute [94, 179, 180, 182, 183]. Recent work by Pine et al. [34] assessed the distribution of OPC-, NPC-, mesenchymal- and astrocyte-like cell states across glioblastoma, IDH-wildtype patient-derived models to determine that no current model could effectively recapitulate all of the cellular states present in this disease and that the dominant states present were dependent on the choice of model. However, transcriptomic analysis determined that the use of cerebral organoids to model glioblastoma produced tumour gene expression signatures significantly more correlated to the original patient tumour than xenografts, 2D cell culture models and 3D tumour cell-only organoids, thus highlighting the potential of this relatively new modelling platform [34]. Moreover, advances in 3D tissue engineering have enabled new approaches to the modelling of gliomas and investigation of novel therapeutics, opening new avenues to bridge the gap between preclinical models and patients. With new and existing modelling platforms having both advantages and disadvantages, understanding the effects of each modelling system on tumour cell biology is crucial (Fig. 4). The development of models that capture our increasing understanding of glioma biology could significantly advance this field and the relevance of preclinical therapeutic development.

### Traditional two-dimensional and three-dimensional cell culture models

Cancer cell line models have been critical to cancer research since their implementation in the 1950s and have contributed greatly to our understanding of cancer biology [184]. *In vitro* cell line models are cheap, typically involve monolayer culture and have enabled the study of molecular mechanisms that would be difficult to elucidate in a full organism [185]. Indeed, *in vitro* monolayer HGG patient-derived cultures have been shown to predict intratumoural clonal population shifts in response to treatment, indicative of the properties of future relapsing tissue [186]. Despite these advantages, typical serum-containing monolayer culture conditions lead to the loss of CSC compartments and the propagation of cells that have vastly deviated genetically and phenotypically from the original parent tumour [94]. Notably, the culture of primary glioma cell lines under serum-containing monolayer culture conditions led to drastic morphological changes whereby cultures with an initially high heterogeneity became homogeneous after one month [94]. Indeed, barcoding experiments have shown that clonal selection occurs in cell lines based on culture conditions and has led to extensive variability between different laboratories culturing the same lines [180]. Screening of 321 anticancer compounds in 27 strains of the MCF7 breast cancer cell line showed that 75% of compounds that were effective in some strains were completely inactive in others, highlighting the unreliability of traditional cell culture models in the context of therapeutic development [180]. Accordingly, transitions to serum-free gliomasphere culture conditions have increasingly been adopted and retain a GSC compartment that more closely imitates parental tumour properties [187]. However, these GSCs have been shown to have higher proliferative character than patient tumour GSCs by scRNA-seq [86]. Given that a primary mechanism of GSC resistance to chemoradiotherapy targeting proliferative populations is quiescence, this represents a critical distinction that may skew results of preclinical models regarding treatment efficacy.

Further shifts towards 3D culture platforms have yielded tumour organoid models that recapitulate the hypoxic gradient, quiescence and heterogeneity seen *in vivo* [34, 188, 189]. These models are typically generated by embedding tumour cells in Matrigel and enabling growth over a period of months [34, 188]. Remarkably, quiescent GSCs have been detected in the hypoxic tumour organoid core, while proliferative GSCs were predominantly found in the outer boundary to demonstrate heterogeneity in GSC populations [188]. Moreover, irradiation of tumour organoids determined decreased apoptosis, as assessed by immunofluorescence imaging of cleaved caspase-3 protein, in SOX2^+^ GSCs compared to SOX2^−^ non-stem tumour cells, recapitulating CSC radioresistance [188]. However, scRNA-seq studies determined that 3D tumour-cell only glioblastoma organoids clustered closer to 2D cell culture models than to xenografts and glioblastoma-cerebral organoids, which clustered closer to the patient tumour [34]. This suggests that the absence of non-tumour cell microenvironmental interactions prevents glioma cells from adopting the relevant transcriptional profiles seen in patient tumours [34]. Nonetheless, these tumour cell-only organoid models harboured a higher fraction of hypoxic cells than any other model, which may prove useful for specific study of these populations [34]. Incorporating non-tumour cells into 3D culture models would thus improve the relevance of cell culture models.

The absence of the non-tumour cell microenvironment results in the loss of critical cell–cell interactions that promote tumour progression and treatment resistance, limiting the relevance of aforementioned *in vitro* models. The relevance of the tumour microenvironment to HGG progression and treatment is strongly reflected by the initiation of a clinical trial (NCT04295759, March 2020) in paediatric HGG patients preventing the microenvironmental release of NLGN3 by ADAM10 inhibition (INCB7839), given that neuronal activity-related NLGN3 release promotes glioma proliferation [131, 190]. Moreover, the formation of TMs known to play a significant role in resistance to surgical resection, chemotherapy and radiotherapy did not occur under *in vitro* glioma spheroid culture conditions, demonstrating a role of the brain microenvironment in TM formation [160, 162]. Accordingly, the relative sensitivities of a TM-high and a TM-low GSC line to temozolomide were reversed *in vitro* compared to *in vivo*, indicative of significant effects of the tumour microenvironment on glioma cell phenotype and therapy response [162]. Furthermore, complete surgical resection of HGGs is impossible due to their diffuse infiltration of the brain parenchyma. Investigating the effects of novel therapies on tissue invasion, not possible in traditional culture methods, would thus be invaluable. As cell culture models lack relevant brain tissue, they are largely unsuitable as a preclinical model and may therefore contribute to trial failure. Improvements to *in vitro* cell culture methods are thus imperative to the development of clinically relevant therapies.

### Animal models

To date, animal models have been undeniably valuable in advancing scientific understanding and therapeutic development across many fields. Animal models circumvent many issues associated with traditional cell culture models, providing a 3D microenvironment for invasion with relevant nutrient and oxygen gradients, as well as complete organ systems. Particularly, murine models have been useful in the study of human biology and disease given that protein-coding regions of human and mouse genomes are 85% identical [191]. Despite these similarities, discrepancies in human and mouse physiology exist that may confound study results. Notably, examinations of neuronal and glial cells in the murine cerebral cortex determined that approximately 55% were glial cells, whereas 92% were glia in the human cerebral cortex [182]. Nonetheless, murine models are widely used and typically involve human patient-derived or cell line-derived xenografts, or the tissue-specific expression of induced tumorigenic mutations typically through Cre-lox recombination engineering methods. Genetically engineered mouse models (GEMMs) are advantageous in that they show site-specific tumour formation in an immune competent animal through the induction of mutations known to initiate human cancers [183, 192]. This enables the study of tumour progression in models that can reflect the genetic and histological heterogeneity in human cancers [183]. GEMMs of high-grade astrocytoma generated by the conditional knockout of *Tp53* and *Pten* tumour suppressor genes in astrocytes resulted in the formation of tumours that acquired additional mutations in RTK genes *Pdgfra*, *Met* and/or *Egfr*, recapitulating human HGG gliomagenesis [193]. Despite these clear advantages, differences in chromosomal arrangements between mice and humans likely affect tumour progression. Moreover, mice lack a true homolog of interleukin-8, known to play a role in GSC maintenance and tumour progression, complicating investigation of therapeutics targeting this pathway [109, 192]. With GEMMs being relatively complex, transplantation of murine or human glioma cells lines, or patient-derived tumour samples, into mice is a relatively common platform. Although these methods model tumour development, the transplantation of established cell lines may carry over aberrations acquired during culture that may influence outcome, or relevant subclones may be lacking. Moreover, while transplants may occur orthotopically in the correct tumour location, often subcutaneous injections occur that lead to the formation of tumours in an irrelevant microenvironment [183]. Additionally, the species mismatch observed in xenografts may impair the supportive communications between tumour cells and the microenvironment to confound results [183]. Species differences may thus impair proper tumour modelling.

Despite species differences, patient-derived orthotopic xenografts are praised for their application in modelling human tumour biology and ability to form heterogeneous tumours specific to a patient. Comparisons of cell states found in paediatric-type diffuse glioma, H3 K27-altered and glioblastoma, IDH-wildtype by scRNA-seq determined that patient-derived xenograft models more closely recapitulated states found in the original patient tumour than glioma sphere and differentiated glioma cell models [34, 39]. With these differences likely attributed to the microenvironment, other work determined that cell culture and *in vivo* conditions differentially activated transcription enhancers to regulate the epigenome and cell state, aligning xenograft tumour transcriptomes closer to the patient tumour [194]. Moreover, murine patient-derived xenograft models were also able to recapitulate differences between cortical and midline paediatric HGGs in terms of interaction with microenvironmental vasculature, with a more intact blood-brain barrier found in midline tumour xenografts, as seen in patients [195]. This thus suggests that the *in vivo* environment provided by animal models has the capacity to appropriately mirror some microenvironmental interactions and the differences in dependencies of different tumour types on particular microenvironmental features. However, xenografts still do not perfectly represent the gene expression signatures apparent in the original patient tumour [34]. Accordingly, examination of H3 and IDH-wildtype, H3 G34-mutant and H3 K27-altered pHGG xenografts determined that these models showed typical HGG histological features, genetic mutations and different cell states found in the parent tumour, but still had a higher proliferative expression signature than the original tumour, as in cell culture models [196]. Moreover, patient-derived orthotopic xenografts have been demonstrated to undergo mouse-specific tumour evolution across 24 different cancer types [179]. Notably, 12 recurrent chromosomal aberrations identified by TCGA for different cancers were lost during xenograft passaging due to different selective pressures in the murine brain microenvironment, including the gain of chromosome 7 and loss of chromosome 10 characteristic of glioblastoma [179]. Given that such aberrations play a role in the development of human tumours, these altered selective pressures may thus lead to inaccurate tumour modelling and alter drug response. Furthermore, xenograft models require host mice to be immunocompromised to allow engraftment of patient tumour cells, thus rendering studies of tumour interactions with the immune system and investigation of immunomodulatory therapies impossible [197]. As such, efforts to develop mouse models with a humanised immune system are underway [197]. Nonetheless, stark differences between tumours under the influence of murine and human microenvironments likely hinder therapeutic development.

### Next generation HGG models - human cerebral organoids

The requirement for preclinical models that accurately reflect HGG tumour interactions in a human microenvironment is apparent. Recent attention has thus focused on 3D modelling of human brain tissues. Human pluripotent stem cells (hPSCs) have been utilised for 3D tissue modelling and are capable of generating most cell types in the human body [198–200]. This term includes both embryonic stem cells (ESCs), which are derived from the inner cell mass of the blastocyst, and induced pluripotent stem cells (iPSCs), which are somatic cells that have been reprogrammed into a pluripotent state by the exogenous application of transcription factors [198–200]. As such, hPSCs have been employed to generate 3D tissues termed cerebral organoids, which contain neuronal and glial cell types organised into discrete brain regions that mirror those seen *in vivo*, as well as an extracellular matrix absent from 2D cultures [201–203]. Typical cerebral organoid culture methods are based on the protocol developed by Lancaster and Knoblich [203]. This involves the generation of embryoid bodies from hPSCs and subsequent neural differentiation of these 3D structures, which are embedded in Matrigel for structural support and expansion [201, 203]. Cerebral organoids have been implemented to study tumorigenesis and invasion of adult glioblastoma, IDH-wildtype tumours primarily by the incorporation of patient-derived tumour cells or the induction of tumorigenic mutations through genetic engineering methods [34, 202, 204–206]. Induction of tumorigenic mutations resulted in the formation of tumours within organoids with a predominantly mesenchymal-like transcriptome, the presence of OLIG2^+^ and SOX2^+^ GSCs and the ability to recapitulate histological features of glioblastoma when implanted into mice [204, 205]. The incorporation of patient-derived GSCs into pre-existing cerebral organoids through gravity-based methods, or dissociation of organoids and reaggregation with tumour cells, has also seen success, given that GSCs are known to re-establish a heterogeneous tumour with the presence of multiple cell states [34, 202, 206]. With this approach more common, numerous studies have identified features of the GSC-cerebral organoid model that recapitulate critical features of HGG pathobiology. Strikingly, the development of TMs following the addition of patient-derived GSCs or primary tumour samples cultured under stem-promoting conditions to cerebral organoids has been observed: a phenomenon absent from other *in vitro* culture methods [162, 202, 206, 207]. These TMs were found at the invasive front of organoid tumours, showed Cx43-positive gap junctions and effectively propagated calcium signals [202, 207]. Moreover, longer TM projections were characteristic of tumour-derived cell lines with increased invasive behaviour and mesenchymal subtype, suggesting that cerebral organoids are capable of reflecting intertumoural heterogeneity of invasive ability [206]. Furthermore, glioblastoma, IDH-wildtype cells showed preference for invasion into the neuronal layers of the cerebral organoid, as opposed to the neural progenitor rosette regions, and did not show invasion of organoids generated from breast cancer cell line MCF10AT or neuroblastoma cell line SH-SY5Y [206]. This suggests that cerebral organoids recapitulate critical features of the brain microenvironment required for HGG invasion and facilitate cell type-specific interactions.

Co-culture of GSCs with mature cerebral organoids mimics microenvironmental signalling pathways observed *in vivo*. The extent of GSC invasion of cerebral organoids has been demonstrated to increase with increasing organoid age [202, 207]. Strikingly, supplementation with synaptic protein NLGN3 (100 nM) increased GSC invasion of 20-day-old organoids to levels comparable to organoids 60 days old, without affecting GSC invasion of 60-day-old organoids [207]. Moreover, immunofluorescence microscopy suggested interactions between GSCs and neurons with synapsin 1-positive presynaptic vesicles in 60-day-old organoids that resembled the formation of hemisynapses [207]. Given that NLGN3 is released from neurons because of neuronal activity and has been demonstrated to promote the growth, invasion and synaptic integration of HGG cells *in vivo*, higher levels of neuronal activity in mature organoids may explain differences in invasive behaviour [32, 131, 132, 207]. This suggests that mature cerebral organoids form a relevant brain microenvironment that can influence GSC phenotypes. Indeed, gene expression analysis of glioblastoma, IDH-wildtype patient-derived tumour cells showed increased expression of genes involved in network formation and invasion, including gap junction alpha-1 (*GJA1*; encoding Cx43) and glypican-3 (*GPC3*), following co-culture with cerebral organoids compared to culture alone [207]. Investigation of the effects of radiation and chemotherapy suggest GSC-derived tumour phenotypes in a cerebral organoid microenvironment are more clinically relevant than traditional *in vitro* culture methods [202]. Irradiation (10 Gy) of a GSC line grown alone in 2D resulted in a reduction in cell viability of 74%, while GSCs cultured with cerebral organoids only showed a 25% reduction in tumour growth, compared to non-irradiated samples [202]. Cytotoxicity assays of alkylating agents temozolomide (1 mM) and bis-chloroethylnitrosourea (BCNU; 100 µM) demonstrated reductions in cell viability for two GSC lines cultured in 2D alone following treatment of greater than 80% and greater than 90%, respectively [202]. Treatment of GSCs cultured with cerebral organoids with temozolomide (1 mM) resulted in reductions in tumour growth of 24% for line 1 and 43% for line 2, while treatment with BCNU (100 µM) resulted in growth reductions of 91% for line 1 and 5% for line 2 [202]. Together, this suggests that GSC-derived tumours have a greater therapeutic tolerance closer to that seen *in vivo* due to the influence of the cerebral organoid microenvironment, and that cerebral organoid models may reflect different chemotherapeutic responses of tumours derived from different patients.

Cerebral organoid models more closely reflect the distribution of cellular states seen in patient tumours than other models [34]. Analysis of scRNA-seq profiles determined that patient-derived GSCs could capture a broader spread of NPC-, OPC-, astrocyte- and mesenchymal-like cell states when cultured with cerebral organoids than in 2D cell culture, as organoids derived from tumour cells only, or in xenograft models, thus reflecting a greater degree of inherent heterogeneity [34]. Furthermore, GSC-derived tumours in cerebral organoids showed a greater proportion of stem-like cells than seen in other models, indicating that cerebral organoid models provide a suitable microenvironment for the support of therapy resistant GSC populations [34]. Additionally, GSC-derived tumours cultured in cerebral organoids showed an enrichment of the proneural transcriptional subtype and OPC- and NPC-like cell states, harbouring an NPC-like subpopulation seen in the primary patient tumour but absent in other models and lost upon re-plating under 2D cell culture conditions [34]. Given that proneural expression signatures have been associated with tumour cells at the outer tumour boundary in contact with neural tissue and brain vasculature, typically left behind during surgery, cerebral organoid models may thus be suited to investigate the effects of therapies on HGG populations that evade resection [95, 96]. However, like other models, cerebral organoid tumours could not perfectly recapitulate the expression signature of the patient tumour, likely owing to the lack of an immune compartment and vasculature [34]. Furthermore, postnatal phenotypes are only acquired in human cortical organoids after 250–300 days in culture, leaving an inability to quickly reach levels of cell type maturity comparable to the adult brain [208]. This lack of cell type maturity alongside the absence of non-neural cell types presents challenges in the implementation of this relatively new modelling platform to investigate HGGs.

### Next generation HGG models - tissue engineering and microfluidic approaches

Advances in 3D tissue engineering have enabled new approaches to the preclinical modelling of diffuse HGG tumours that recapitulate the interactions of tumour cells with features of the native human brain microenvironment. Engineering tissues for cancer modelling presents potential to control and define cell types present and the surrounding 3D microenvironment, facilitating improvements in reproducibility and the ability to design a microenvironment conducive to the maintenance of diverse tumour subpopulations. The engineering of tissues through 3D bioprinting involves the distribution of cells within a bioink composed of natural, synthetic or semisynthetic biomaterial that is then printed according to a design developed through computer aided design (CAD) software [209]. This allows precise control over the spatial distribution of cells and their surrounding microenvironment for more reproducible 3D tissue generation and effective modelling not previously possible [209]. Notably, Neufeld et al. [35] developed a glioblastoma bioink comprised of patient-derived glioblastoma cells, human astrocytes and human microglia dispersed in fibrinogen and gelatin in an attempt to mimic the complexity of tumour cell interactions with the surrounding brain microenvironment and extracellular matrix (ECM). Indeed, this 3D approach produced differences in growth rate and invasion similar to those seen *in vivo* between dormant and fast-growing glioblastoma tumour clones, whereas two-dimensional monolayer culture produced no differences in growth patterns and invasive capabilities, further supporting the notion that monolayer culture abolishes heterogeneity [35]. Moreover, this translated to the 3D bioink modelling differences in the effects of temozolomide on different patient-derived glioblastoma cells, producing ranges in the IC_50_ values from 280 to 1400 µM, whereas monolayer culture produced IC_50_ values ranging from 0.004 to 0.005 µM [35]. This suggests that the inclusion of non-neoplastic cell types in a 3D microenvironment facilitates the recapitulation of differences in the responses of patients to therapeutics, as well as relevant survival cues that increase therapeutic tolerance to levels more similar to those found *in vivo*.

Additional 3D bioprinted constructs have demonstrated the necessity for the inclusion of non-neoplastic cell types and appropriate ECM for relevant HGG modelling. Tang et al. [210] bioprinted human GSCs, astrocytes and NPCs with or without macrophages in a gelatin methacrylate (GelMA) and glycidyl methacrylate-hyaluronic acid (GMHA) hydrogel. This enabled the recapitulation of the high hyaluronic acid content of the brain microenvironment and the potential to control gel stiffness through methacrylate cross-linking [210]. Physiologically relevant cues were able to be mimicked by precise printing into a tumour core region containing GSCs with or without macrophages, and a surrounding peripheral region comprised of astrocytes and NPCs with a compressive modulus of 0.9 ± 0.2 kPa akin to healthy brain tissue, reported at ~ 1 kPa [210]. The resultant transcriptional profile of GSCs grown in the 3D bioprinted gels displayed stronger similarity to glioblastoma patient tumour specimens than GSCs grown in sphere culture, as well as an upregulation of GSC- and mesenchymal-subtype-related genes [210]. Moreover, the presence of macrophages in printed scaffolds produced increased expression of hypoxia- and invasive-related genes compared to scaffolds lacking macrophages, indicating that interactions with individual cell types were influential [210]. This implementation of 3D bioprinting techniques has enabled the inclusion of microglia and macrophages typically lacking in other models, allowing studies relating to interactions with the immune compartment and treatment efficacy to be investigated. With regards to treatment efficacy, significant differences in cell viability were observed between 3D bioprinted GSCs and GSCs grown in sphere culture when treated with temozolomide, erlotinib and gefitinib, with bioprinted co-culture constructs typically showing higher therapeutic resistance [210]. Indeed, a higher therapeutic tolerance of GSCs treated with temozolomide has been reported when 3D bioprinted in gelatin/alginate/fibrinogen hydrogel compared to 2D culture by up to two-fold [211]. These differences in treatment efficacy arising from the addition of non-neoplastic cell types and a supporting hydrogel highlight that the choice of model for drug screening influences study outcome, with 3D bioprinted models bearing greater resemblance to the therapeutic resistance observed in HGG patient populations compared to traditional cell culture models.

The importance of vasculature in the pathobiology of HGG tumours has been recognised in 3D bioprinting applications. Wang et al. [212] implemented coaxial extrusion bioprinting, involving a sheath/core nozzle comprised of two concentric circles, to generate an inner core consisting of human umbilical vein endothelial cells (HUVECs) printed in a collagen gel with an outer layer of human glioblastoma (U118) cells encapsulated in a calcium-crosslinkable sodium alginate solution. The co-culture of HUVECs and U118 resulted in higher relative cellular proliferation than observed in the culture of either cell type alone, as well as an increase in the formation of HUVEC tubule-like structures, suggestive of angiogenic activity, than in the culture of HUVECs alone [212]. This suggests that cell-cell signalling between HGG and endothelial cells in the 3D bioprinted constructs is influencing cell phenotype and, although not investigated in the study, could prove useful in investigating the effects novel therapeutics on perivascular glioma cells and angiogenesis/vessel co-option. Further 3D bioprinting work generated a perfusable vascular network within a bioink comprised of human glioblastoma cells, astrocytes and microglia in fibrinogen and gelatin [35]. A layer of the cellular fibrinogen and gelatin bioink was printed before a sacrificial bioink composed of Pluronic F127 and thrombin was overlayed in a network design [35]. Following the addition of another cellular fibrinogen and gelatin layer, the sacrificial bioink was evacuated and HUVECs and human pericytes were added to the hollow channel to form a vascular lumen [35]. Studies involving 70 kDa dextran-FITC demonstrated that this HUVEC and pericyte-containing vascular network was perfusable, while confocal microscopy revealed that this network existed within the human glioblastoma, astrocyte and microglia-containing bioink [35]. This overcomes some of the limitations associated with previous models, as this glioblastoma model contained perfusable blood vessels absent from other *in vitro* platforms, as well as other cell types within the brain. However, neurons were lacking from this printed platform, likely due to difficulties associated with the printing of mature neurons typically circumvented by printing NPCs. These studies highlight the degree of spatial control provided by 3D bioprinting that facilitates better design and fabrication of relevant 3D brain microenvironments, which may be implemented to improve current HGG modelling platforms.

Additional advances in 3D tissue engineering that may assist in improving the clinical relevance of models include the application of microfluidic devices. Microfluidic devices are systems that involve the movement of small amounts of liquid through channels on a chip that have recently been implemented to mimic the dynamics of the tumour microenvironment, including nutrient gradients and drug delivery [213]. These systems, typically termed tumours-on-a-chip, are microfabricated by techniques including photolithography and soft lithography and involve the transfer of a pre-designed pattern to the chip to result in the desired arrangement of microchannels, to which cells may be added [214]. These devices may be connected to an external reservoir to deliver culture media or other compounds in a time- and concentration-dependent manner [215]. Microfluidic and 3D bioprinting technologies were employed by Silvani et al. [216] to generate a glioblastoma-on-a-chip model. A GelMA-fibrin bioink loaded with human brain endothelial cells was 3D bioprinted into a ring shape and a GelMA-alginate bioink containing human glioblastoma cells was subsequently printed into the internal core region [216]. A surrounding flow channel was coated with fibronectin to allow adhesion of human brain endothelial cells or HUVECs to the channel walls and was subjected to physiologically relevant shear stress of 9 dyne/cm^2^ by perfusion with growth media [216]. This resulted in the endothelial cells organising themselves into a cylindrical functional barrier that prevented the diffusion of fluorescent dextran across the wall, with brain endothelial cells showing higher resistance, likely due to their innate blood-brain barrier functions [216]. Thus the application of microfluidic devices and potential to induce shear stress by flow may be advantageous over other models through better recapitulation of cell phenotypes, given that blood flow is critical in vessel wall maturation and remodelling both *in vitro* and *in vivo* [217]. Although the aforementioned glioblastoma-on-a-chip model did not investigate therapeutic effects or cell-cell interactions, it highlights the utility of microfluidic platforms to provide physiologically relevant features that may improve clinical relevance, particularly in combination with the spatial control offered by 3D bioprinting techniques.

Additional studies have highlighted the capacity for microfluidic systems to model cell-cell interactions and enhance therapeutic development, although they did not implement the potential for continuous flow. Truong et al. [218] demonstrated the physiological relevance of a microfluidic system in the modelling of GSC interactions with the surrounding vascular niche, with increased GSC invasive potential through CXCL12-CXCR4 signalling recapitulated. HUVECs within a fibrin hydrogel were injected into the vascular channel of the device, and patient-derived GSCs within Matrigel were injected into the tumour region, with Matrigel added to the stroma-like region between them [218]. GSCs in the presence of HUVECs were shown to have a significantly higher migration distance compared to GSC-only microfluidic devices, which was shown to decrease in a dose-dependent fashion with the addition of CXCR4 antagonist AMD3100 (0 – 100 µM) [218]. Recent work from Straehla et al. [219] also implemented a microfluidic vascularised glioblastoma model, with an aim to improve the development of nanotherapeutics targeted to tumour associated vasculature. Spheroids comprised of human glioblastoma, IDH-wildtype cells and human brain pericytes were surrounded in fibrin gels containing human PSC-derived endothelial cells, human brain pericytes and astrocytes and injected into microfluidic devices for vascularisation over 7 days [219]. Transport receptor LRP1 at the blood-brain barrier, shown to be upregulated in glioblastomas, was also upregulated in vessels in the microfluidic model in the presence of glioblastoma cells, compared to vessel-only controls, and was also present in glioblastoma cells [219, 220]. Functionalisation of nanoparticles with AP2 peptide, a LRP1 ligand, enabled targeting to LRP1, as demonstrated by microscopy [219]. Transport of nanoparticles from the abluminal to the luminal side of the vessels was subsequently shown to occur by transcytosis via LRP1-mediated active transport, with this permeability significantly decreased by antibody neutralisation of LRP1 [219]. Moreover, the encapsulation of cisplatin in the liposome core of AP2-functionalised nanoparticles increased the efficacy of cisplatin in the microfluidic device model [219]. This was demonstrated by increased expression of caspase-3 in tumour tissue by qRT-PCR and significantly higher Sytox signal, a nucleic acid stain for dead cells, at the glioblastoma tumour region, compared to regions far from the tumour, to non-functionalised or non-loaded nanoparticle controls, and to free-cisplatin treated controls [219]. Matching orthotopic murine xenografts treated with cisplatin-loaded AP2-functionalised nanoparticles also showed slower tumour growth compared to those treated with free cisplatin, thus suggesting that the microfluidic device could model *in vivo* properties related to transcytosis and nanoparticle targeting [219]. Thus, these microfluidic systems may be implemented as effective blood-brain barrier models to investigate the effects of nanotherapeutics as well as interactions of HGG cells with vasculature in an *in vitro* human platform.

Further studies employing microfluidic devices highlight their potential to recapitulate tumour heterogeneity and known properties of tumour subtypes, as well as improve the clinical relevance of therapeutic development. Xiao et al. [221] encapsulated fluorescently labelled HUVECs and glioblastoma cells in a 3D fibrin gel that was pipetted into a central microchannel, surrounded by two flow channels for culture medium [221]. Analysis of confocal microscopy images determined that patient-derived GSCs showed significantly higher colocalisation with the microvasculature than the U87 glioblastoma cell line, suggesting that the perivascular niche model supports GSCs as seen *in vivo*, and may facilitate the “homing” of particular cell subpopulations to the vasculature [221]. Moreover, single-cell RNA-sequencing of 10 patient-derived cell lines incorporated into the microvasculature-on-a-chip model determined that proneural, invasive mesenchymal and stem-like gene expression signatures were correlated with increased vasculature colocalisation [221]. This suggests that the microfluidic platform recapitulates the dependencies of these cell subpopulations on the vasculature and supports the maintenance of diverse phenotypes, as required for accurate preclinical modelling and lacking from traditional cell culture methods.

Furthermore, Yi et al. [36] demonstrated that *ex vivo* patient-derived glioblastoma-on-a-chip models reflected patient-specific resistances to chemoradiotherapy, harnessing biophysical and biochemical cues made possible by the microfluidic device. A bioink was generated from porcine brain decellularized extracellular matrix (BdECM) in an attempt to better reflect the complexity of the native brain ECM than other bioinks [36]. HUVECs were encapsulated in the BdECM bioink and printed in a ring structure, which was then filled with BdECM bioink laden with glioblastoma cells [36]. These 3D cell structures were surrounded by a gas-permeable silicone ink and covered top and bottom by a gas-impermeable glass substrate, resulting in a radial oxygen gradient as gas was only available through the silicone wall [36]. Thus, hypoxia was evident at the glioblastoma core and vascularised stroma surrounded the tumour core, reflecting two critical microenvironmental features associated with HGG pathobiology [36]. Strikingly, this arrangement resulted in the emergence of SOX2^+^-therapy resistant glioblastoma cells, which were completely lacking in chip controls where there was no oxygen gradient or when HUVECs and glioblastoma cells were mixed in the bioink, removing compartmentalisation [36]. Moreover, glioblastoma cells derived from patients with low to moderate treatment resistance (Group X), high treatment resistance (Group Y) and extremely aggressive progression following treatment (Group Z) applied to the microfluidic platform and treated with concurrent radiotherapy (15 Gy) and chemotherapy (temozolomide, 950 μM) recapitulated the differences in response observed in the clinic [36]. Glioblastoma cells from Group Z patients showed significantly higher levels of survival following treatment in the microfluidic platform than Group Y cells, with both significantly higher than Group X, as assessed by a CCK-8 cell viability assay [36]. Of particular clinical note, primary cells harvested from Group Z patients before patient exposure to temozolomide chemoradiotherapy and after patients were treated showed differences in treatment response in the microfluidic platform, which was absent from monolayer culture and spheroid culture systems and reduced in the microfluidic platform lacking the oxygen gradient [36]. Cells harvested after patient exposure to chemoradiotherapy showed a significantly higher survival following treatment in the microfluidic device, suggesting that the microfluidic platform was the only model studied capable of reflecting the increased resistance of tumour cells to therapy following treatment [36]. This thus indicates that the more parameters added that reflect the tumour microenvironment, the closer preclinical trial results will be to what is observed in patient cohorts.

The addition of such microenvironmental features not possible in other cell culture models through the use of 3D bioprinting and microfluidic techniques thus supports cellular heterogeneity and the emergence of therapy-resistant populations, thereby achieving more clinically relevant outcomes. However, the preparation of bioinks for printing or casting into devices typically involves the encapsulation of already differentiated cell types, of different genetic background and developmental stage, in order to achieve the aforementioned cell type complexity [35, 210, 219]. In doing so, potential to recapitulate the complex tissue architecture, cell patterning and self-organisation into distinct brain regions seen during normal embryonic development is lost. Moreover, current cell culture protocols for microfluidic devices are not yet standardised and the surface chemistry of devices may differ, each leading to potential for variability in results [222]. Thus, further improvements to these promising new technologies are still required in the search for clinically relevant models.

### Potential improvements to next generation models to attain clinical relevance

With the benefits and limitations of current models clear, potential avenues for their improvement and further development surround advances in tissue engineering and PSC-related technologies. One platform that may be employed to improve the maturity of cell types within cerebral organoids, and hence the design of better HGG models, is electrical stimulation. The premise of electrical stimulation in tissue engineering is to alter cell fates and phenotypes, drawing on principles of endogenous electrical signals that are known to play vital roles in cellular and tissue functions through the modulation of gene expression, cell migration, morphology and cell-cell signalling [223–227]. Given the central role of electrical signals in the nervous system, numerous studies have characterised the influence of exogenous electric fields on neural tissue in enhancing maturation and neuroregeneration through mechanisms that include altering gene expression and neurotransmitter release [226]. Neurons in an exogenous electric field experience transmembrane potential perturbations that open voltage-gated ion channels to trigger calcium influx, activating calcium-dependent enzymes that increase transcription of genes involved in neuronal differentiation, survival and synaptic plasticity [226]. Electrical stimulation (± 0.25 mA/cm^2^, biphasic waveform of 100 μs pulses with 20 μs interphase, 250 Hz, 8 h per day for 3 days) of human NSCs (hNSCs) has been demonstrated to direct cell fate towards βIII-tubulin (Tuj1)^+^ neurons with a reduced induction of GFAP^+^ glial cells; to increase neurite length; and to induce the formation of neuronal clusters interconnected by neurite networks [224]. Moreover, Tomaskovic-Crook et al. [225] demonstrated that the electrical stimulation of hNSCs encapsulated in a conductive 3D biogel resulted in an increased ratio of mature neuronal marker MAP2 to immature neuronal marker Tuj1 in stimulated compared to unstimulated controls [225]. Further, calcium imaging determined an increase in firing rate and calcium flux amplitude following disinhibition of cells with GABA receptor-A antagonist bicuculline for stimulated constructs not apparent in unstimulated controls [225]. These results reflect an enhanced maturity and function of neural networks that contain inhibitory GABAergic neurons [225]. Electrical stimulation may therefore overcome limitations associated with an inability to reach levels of cell type maturity in organoid cultures reflective of the brain *in vivo* and lead to the design and implementation of more clinically relevant models.

Electrical stimulation has also been demonstrated to modulate the fate of non-neural cell types [227–229]. Electrical stimulation (± 0.1 mA/cm^2^ for 8 h per day for 3 days, followed by ± 0.25 mA/cm^2^ for 8 h every 2 days for 6 days) of human iPSCs has been demonstrated to reduce the expression of pluripotency markers and increase the expression of endodermal, mesodermal and neuroectodermal markers by qRT-PCR [227]. Strikingly, single electrical field pulses (250–750 V m^−1^, 60 s) have also been shown to dose-dependently increase the area of CD31^+^ capillary-like structures in murine embryonic stem cell (mESC)-derived embryoid bodies and VEGF expression, compared to unstimulated controls, suggesting enhanced endothelial cell maturation and angiogenesis [228]. Additionally, electrical stimulation (200 mV mm^−1^, 24 h) of HUVECs has been demonstrated to increase VEGF release, as well as increase endothelial cell elongation and alignment *in vitro* in a direction perpendicular to the applied field vector [230]. This reorientation required VEGF receptor (VEGFR) signalling, with pharmacological inhibition of VEGFR (4-[(4′-chloro-2′-fluoro)phenylamino]-6,7-dimethoxyquinazoline, 50 µM) preventing alignment [230]. Electrical stimulation may thus enhance the maturation of a variety of tissues, including hPSC-derived vascularised cerebral organoids.

An additional limitation of the application of organoids to HGG modelling is the inherent variability between organoids, owing to the inability to completely control their self-assembly and cell-cell signalling [231]. Differences in the properties and composition of replicate organoids may lead to variations in the responses of glioma cells to novel therapeutics, given that cells in the surrounding brain microenvironment have been demonstrated to affect glioma cell phenotype. Established protocols for the generation of human brain organoids may also contribute to variability and typically involve the encapsulation of 3D hPSC-derived aggregates in Matrigel [201, 203]. Matrigel itself is a gelatinous mixture of ECM proteins derived from Engelbroth-Holm-Swarm murine sarcomas that lacks chemical definition and exhibits batch-to-batch variability, which may influence culture outcomes [232–234]. Accordingly, recent work has been shifting towards the culture of organoids without Matrigel into more chemically defined, controllable systems including GelMA and recombinant protein matrices [235–237]. Indeed, Tomaskovic-Crook et al. [237] demonstrated the reproducible generation of dorsal forebrain organoids on GelMA hydrogels with the defining cortical tissue architecture and electrophysiological activity. In addition to the benefits of chemical definition, the ability to photocrosslink hydrogels such as GelMA in the presence of a photoinitiator due to its methacrylation enables the fine tuning of the elasticity of the surrounding microenvironment, which is known to influence cell fate and phenotype [238–240]. A higher degree of substitution, polymer concentration, photoinitiator concentration and photocrosslinking time may each increase the mechanical stiffness of GelMA hydrogels [239, 241]. This ability to control mechanical properties serves as an advantage over Matrigel, which has a variable elastic modulus reported between 0.12 and 0.45 kPa [242]. Transitions to more defined and reproducible culture methods may thus limit organoid variability and improve the application of cerebral organoids to HGG modelling.

HGG cells have been demonstrated to interact heavily with neighbouring vasculature (as detailed above), reinforcing the need for vascular cell-cell interactions to be present in models for the accurate assessment of new therapies. Although the bioprinting of vascular cells alongside glioma cells has been discussed, the application of vascularised cerebral organoids to HGG modelling would also be advantageous to enable the study of HGG-vascular interactions alongside neural tissue. However, the development of methods for the vascularisation of cerebral organoids that would be relevant to HGG modelling appears challenging. Methods to date have primarily involved the incorporation of HUVECs or other endothelial cell types into hPSC-derived organoids, implantation of hPSC-derived organoids into mouse brains, lentiviral-based methods of ectopic expression or the fusion of neural and mesodermal spheroids [243–247]. Mansour et al. [243] engrafted hPSC-derived cerebral organoids into murine brains to demonstrate infiltration of host CD31^+^ vessels and blood flow to the organoid. Although this method would allow the establishment of endothelial cell interactions and nutrient gradients within human cerebral tissue, such a model is still limited in that vasculature is mouse-derived and cross-species interactions in an inappropriate microenvironment exist.

Alternatively, Shi et al. [245] incorporated HUVECs into hPSC-derived neural organoids during initial stages of embryoid body formation, subsequently demonstrating the formation of extensive vascular networks expressing laminin and isolectin I-B4. HUVECs cultured in cerebral organoids showed expression of the ABCB1 drug efflux pump, known to be present on endothelial cells forming brain capillaries, whereas HUVECs cultured alone showed no expression, suggesting HUVECs can adopt a more relevant phenotype in the organoid microenvironment [245]. However, differences between HUVECs and brain microvascular endothelial cells (HBMECs) exist that reflect their differences in function [248, 249]. HBMECs express higher levels of occludin (OCLN) and zonula occludens-1 (ZO-1) proteins than HUVECs, which are involved in the formation of tight junctions between endothelial cells [249]. Since the integrity of the blood brain barrier requires the presence of tight junctions between endothelial cells and lower permeability than in other vascular regions, differences in the expression of these proteins is unsurprising [248]. Accordingly, dextran perfusion experiments determined that microvasculature derived from HBMECs had a threefold lower permeability than that derived from HUVECs [249]. Whether culture with hPSC-derived cerebral organoids alters the expression of these proteins in HUVECs is unclear but requires consideration in the establishment of relevant models. Moreover, application of vascularised cerebral organoids to study HGG requires modelling of the invasive properties of HGG cells along vasculature and neuronal tracts [250]. Given that HUVECs will have a different genetic background to hPSC-derived organoids, differences in the migration ability of HGG cells along neuronal tracts and blood vessels cannot be attributed to cell type alone, limiting relevance. Additional cell types involved in the proper formation and function of blood vessels and the perivascular niche are also absent in HUVEC-based models, including pericytes, which may also limit applicability. Song et al. [246] formed vascular spheroids from hPSC-derived NPCs and hPSC-derived endothelial cells, with the addition of mesenchymal stem cell (MSC) lines [246]. Although MSC-derived cells can function as pericytes, such models are still hindered by variance in genetic background [246]. With brain vasculature a preferred migration route for invading glioma cells, generating appropriate models for these interactions is critical [139, 250].

The derivation of hPSC-derived vascularised cerebral organoids with uniform genetic background has also been described [244, 247]. Cakir et al. [244] ectopically expressed Ets variant 2 (ETV2) in hPSCs by transfection with a doxycycline-inducible lentivirus containing *ETV2*, known to induce endothelial cell differentiation. Transfected hPSCs were mixed with non-transfected parental hPSCs to generate vascularised cerebral organoids, forming a CD31^+^, ZO-1^+^ and OCLN^+^ vascular network that could form functional vessels and connect to host vasculature following subcutaneous implantation into mice [244]. Further, the presence of endothelial cells was associated with enhanced functional maturity of neurons, as demonstrated by an increased proportion of neurons generating action potentials compared to cells in non-vascularised organoids by patch-clamp recordings [244]. Considering that communication between neural and vascular tissue is critical for the proper formation of the CNS, as neural and vascular structures develop and mature concomitantly, an enhanced maturity during co-culture is expected [251]. Despite these advantages, the requirement for doxycycline to induce controlled *ETV2* expression may be problematic, given than doxycycline has been demonstrated to enhance the self-renewal of hPSCs and hNSCs, as well as dopaminergic neuronal differentiation [252, 253]. This may alter the cell fate of non-transfected hPSCs and affect the ability to generate organoids representing specific brain regions. Alternatively, Worsdorfer et al. [247] generated vascularised cerebral organoids by the fusion of hPSC-derived neural and mesodermal spheroids, giving rise to neural as well as endothelial, mural and immune cell types. The CD31^+^ endothelial network was shown to infiltrate the neural region of the assembled organoid, with associated α-smooth muscle actin (αSMA)^+^ mural cells and ionised calcium-binding adaptor molecule 1 (IBA1)^+^ macrophage- or microglia-like cells [247]. Moreover, vessels were functional following implantation into chicken embryos [247]. Although these are promising features, this method led to the generation of organoids with clearly separate mesodermal and neural parts, which is not reflective of brain structure *in vivo* and thus not appropriate for modelling HGG invasion of brain tissue. Thus, improvements to these protocols are imperative prior to their application to HGG modelling.

Although tissue engineering techniques have circumvented some issues associated with the need for a variety of cell types to be present for relevant HGG modelling, printing cell types within a gel is still not reflective of native brain microenvironments. The ECM of the brain is synthesised by neurons and glial cells and is composed of a complex mixture of glycosaminoglycans, proteoglycans, glycoproteins and low amounts of fibrous proteins, such as collagen [254]. Aforementioned bioinks are primarily collagen- or fibrinogen-derived, not reflective of the major brain ECM components important to HGG modelling, with fibrinogen not typically found in the CNS [255]. The design of a bioink that incorporated all of the relevant ECM features would be challenging. To overcome these limitations, recent studies have developed hydrogels from BdECM derived from porcine and human brains [36, 256]. Although species differences between porcine and human brain tissue may be limiting, patient-derived glioblastoma cells demonstrated increased invasion and an elongated morphology when cultured in the BdECM gel compared to a collagen gel, thus demonstrating the importance of the choice of encapsulating biomaterial in reflecting cell phenotypes [36]. However, since BdECM may be derived from individuals and the decellularisation of tissues and modifications for gel formation are necessary, variability between gels may be introduced at a variety of steps [36, 256, 257]. Hence, the design and standardisation of bioinks better reflective of the native brain microenvironment is still necessary.

The choice of cell type for use with 3D tissue engineering strategies also shapes the relevance and potential of resultant tissues. As described above, fully differentiated cells of different genetic background are often used for 3D tissue engineering purposes [35, 210, 219]. This results in an inability to accurately model features of development that result in the complexity of tissue structure seen *in vivo*. Mirroring such features may be important, given that glioblastoma, IDH-wildtype cells showed preference for neuronal layers over progenitor regions in cerebral organoids for invasion [206]. Moreover, given that certain paediatric HGGs are characterised by somatic histone mutations, these diseases are thought to arise due to epigenetic influences during neurodevelopment [51, 54, 55]. The use of already mature cell types thus removes the potential to study such influences in the aforementioned models, with neurodevelopmental timing of particular cell-cell interactions likely to be of importance. Furthermore, although the application of HUVECs and brain endothelial cells to tissue engineering platforms has been demonstrated to result in the formation of tubular structures, these lack the cell type complexity of vasculature *in vivo*, where there are surrounding pericytes and vascular smooth muscle cells that form functional tissue [212, 216]. However, this has been recapitulated in tissues derived from PSCs, where pericytes and/or smooth muscle cells and astrocytes have been demonstrated to associate with endothelial cells, reminiscent of vasculature and the blood-brain barrier [247, 258, 259]. This highlights that although 3D tissue engineering technologies have enabled precise spatial control and a variety of vascular, immune and neural cells types to be present, they do not reflect native tissue development. Although the application of PSCs to generate organoids better reflects features of embryogenesis, it is particularly challenging to generate the complexity of cell types required, with neural, vascular and immune cell types arising from different germ layers, and the ability to precisely control structure and organisation is lacking. Thus, the development of clinically relevant models will likely require additional advances in and the combination of PSC and 3D tissue engineering technologies.

Improvements regarding PSC-derived tissues with diverse compartments reflecting different tissue regions or derived from different germ layers are underway, as well as the application of PSCs in 3D tissue engineering techniques. The generation of cortico-motor assembloids by the fusion of hPSC-derived cortical organoids, hindbrain/spinal organoids and skeletal muscle spheroids and their subsequent culture demonstrates the potential of rapidly advancing PSC-culture techniques [260]. Strikingly, optogenetic or chemical stimulation of the cortical region of the assembloid was communicated through the hindbrain/spinal region to result in skeletal muscle contractions, thus demonstrating the potential of 3D cultures to form functional tissue models [260]. Assembly of neural and mesodermal or vascular spheroids has also been demonstrated to produce vascularised brain organoids, although these approaches are limited by the remaining separation of neural and vascular compartments, as well as the inability of vascular tissues to reach maturity in the absence of shear stress generated by flow [246, 247, 258]. Nonetheless, these approaches highlight the feasibility of PSC technologies to generate more advanced tissues. Moreover, the incorporation of PSCs into 3D bioprinting techniques has been demonstrated. The formation of functional neural tissues containing neurons with and without glia has been reported by the 3D bioprinting of hPSCs and hPSC-derived neural progenitors in fibrin, alginate and/or Matrigel-based inks [261–263]. Not only does this provide potential to model some aspects of *in vivo* neurogenesis and patterning, but it also facilitates the derivation of different cell types with the same genetic background, enabling the subsequent interactions with glioma cells observed to be attributed to cell type rather than variation in genetic background of sourced cell lines. This combination of spatial control offered by 3D bioprinting and the developmental potential of hPSCs will likely prove advantageous in tissue modelling platforms. Furthermore, hiPSC-based technologies allow potential for modelling platforms to be patient-specific, leading to the possibility for more personalised medicine, although generating patient-specific iPSC models using current technologies requires time investments that may not be feasible in the clinic. Thus, further advances to current technologies will likely improve the relevance of modelling platforms.

The combination of 3D printing, biomaterial, microfluidic and PSC-derived organoid technologies will likely lead to improvements in *in vitro* modelling platforms that translate to more clinically relevant outcomes. Cho et al. [256] demonstrated that the application of microfluidic devices and BdECM to cerebral organoid culture improved organoid maturity in a reproducible manner, overcoming some aforementioned limitations associated with PSC-based techniques. A microfluidic platform consisting of two chambers for organoid culture and three chambers as medium reservoirs connected by microchannels was implemented, with fluid flow in the platform achieved by placing the device on a bi-directional rocker to generate hydrostatic pressure [256]. While encapsulation in BdECM alone led to the generation of larger cerebral organoids compared to Matrigel or decellularised ECMs from other organs, indicative of BdECM providing specific cues that better support cerebral organoid generation, application of the microfluidic devices led to further increases in organoid size, as well as increased proliferation and decreased apoptosis, as assessed by immunostaining for Ki67 and caspase-3 expression [256]. This corresponded to significantly higher and more uniform levels of oxygen within organoids cultured in the device compared to BdECM-only controls, as assessed by oxygen-sensing phosphor nanoparticles [256]. This overcomes a significant limitation of organoid culture regarding their limited size and the formation of a necrotic core, which arises due to the inability of oxygen and nutrients to reach the core under standard culture conditions. Moreover, culture in the microfluidic platform led to improved cortical layer development and electrophysiological function, as well as a higher presence of microglia, as assessed by immunocytochemistry, calcium imaging and patch clamping [256]. Furthermore, qPCR analysis demonstrated less variability in gene expression profiles between organoids cultured in the microfluidic platform compared to BdECM-only controls [256]. Together, this suggests that the application of microfluidic devices to cerebral organoid culture techniques may improve the maturity and reproducibility of resultant organoid cultures, as well as support the presence of an immune compartment.

Recent work by Salmon et al. [264] applied microfluidic, organoid and 3D printing techniques to generate a neurovascular organoid model-on-a-chip. A microfluidic device with a central organoid chamber flanked by microchannels for vascular cell seeding was designed and manufactured with CAD software and stereolithography 3D printing [264]. Pericytes and endothelial cells were subsequently differentiated from hPSCs and added to the microchannels for co-culture with hPSC-derived cerebral organoids in the central chamber [264]. At days 20 and 25 of culture, the device was connected to a syringe pump and perfused with 1 μm red fluorescent beads that demonstrated the functional barrier formation of the vascular cells, as the beads did not leak into the surrounding gel as observed for the 40 kDa dextran [264]. Thus, the application of microfluidic devices enables vascular assessment and also provides potential to enhance the maturity of hPSC-derived vascular structures through applied flow rates, mimicking blood flow [217, 264]. Despite combining these techniques, endothelial cells only reached the core of 42% of organoids [264]. Although not yet perfect, this is still an improvement over previous organoid vascularisation attempts and highlights the potential of these 3D tissue engineering techniques to provide more relevant modelling platforms for the study of HGGs as well as other diseases.

## Conclusions and future directions

Diffuse HGGs have low survival rates and lack curative treatment options. Established modelling platforms do not reflect the human brain microenvironment that diffuse HGG tumour properties depend on, altering the tumour cell phenotypes present to likely skew preclinical studies and contribute to poor oncology clinical trial success rates. Microenvironmental cues are known to influence cell phenotypes, with perivascular, hypoxic and invasive niches known to promote the maintenance of therapy resistant GSC subpopulations. Moreover, recurrent associations of transcriptional subtypes with regional and structural features further support microenvironmental influence, with tumour cell interactions with non-neoplastic cells including endothelial cells, TAMs and neurons demonstrated to drive tumour proliferation and alter gene expression. The absence of an appropriate microenvironment results in cell state distributions and therapy responses that deviate from patient tumours, likely leading to the high attrition rates of preclinical anticancer agents. Indeed, disparities between the responses of tumour cells to therapy when grown under different conditions have been established, highlighting the importance of assessing tumour cells in appropriate microenvironmental contexts to attain clinically relevant findings.

Recent shifts towards human 3D tissue modelling platforms promise to improve relevance by mimicking the human brain microenvironment and enabling multi-directional communication between neoplastic and non-neoplastic cells. The potential to generate 3D brain tissues with PSC-based techniques, as well as the application of 3D bioprinting, microfluidic devices and other emerging tissue engineering techniques, presents avenues for the development of *in vitro* platforms that enable interrogation of cause and effect in human tissues, circumventing issues associated with species differences in animal models and homogeneity of monolayer culture. Based on the literature discussed in the sections above, the authors recommend that improvements to *in vitro* modelling platforms to attain clinical relevance should be implemented as follows:

- hPSC- and organoid-based techniques should be further advanced to enable the presence of neural, vascular and immune compartments arising from different germ layers within the one tissue, enabling side-by-side development and maturation. Improved modelling of features of embryogenesis could result in the production of tissues more akin to the native brain microenvironment than can be achieved by co-culture of pre-differentiated cell types. This would support the maintenance of HGG cell subpopulations in their appropriate niche environments for closer modelling of the effects of novel therapies and interrogation of resistance mechanisms in particular populations.
- 3D bioprinting should be implemented to overcome limitations associated with a lack of precise spatial control in organoid cultures. This could be achieved by printing hPSCs and/or early hPSC-derived progenitors to allow side-by-side maturation. It could also involve the 3D bioprinting of HGG cells onto 3D tissues to control their distributions and site of attachment. This spatial control may improve the design and reproducibility of PSC-derived tissue models for HGG, enabling consistent and relevant results.
- Electrical stimulation should be applied to overcome the limitations associated with cell type immaturity in PSC-derived tissues.
- Biomaterials for encapsulation of tissues more reflective of the appropriate native ECM should be selected. For HGG cells, this would also enable invasive phenotypes to be more accurately interrogated.
- The potential of microfluidic devices to improve the relevance, reproducibility and maturity of models by mimicking the dynamic state of tissues should be harnessed. These improvements could be realised by these devices increasing the availability of nutrients to organoids and introducing flow to support growth, maturation, and consistency, as well as by the application of the devices to recapitulate oxygen gradients and tissue compartmentalisation for HGG tumour cells.

Although these recommendations carry an increased cost burden, so does continuous preclinical study failure. Future research should thus strive to develop modelling platforms that recapitulate as much of the human brain microenvironment and intratumoural diversity as possible by implementing the above recommendations combining PSC-based, 3D bioprinting, electrical stimulation and microfluidic techniques. This would best apply our increasing understanding of HGG pathobiology to enable the development of more clinically relevant therapeutic strategies, thereby improving patient outcomes.

## References

[1] Ostrom QT, Cioffi G, Gittleman H, Patil N, Waite K, Kruchko C (2019). CBTRUS Statistical Report: Primary Brain and Other Central Nervous System Tumors Diagnosed in the United States in 2012–2016. Neuro-Oncology.

[2] Louis DN, Perry A, Wesseling P, Brat DJ, Cree IA, Figarella-Branger D (2021). The 2021 WHO Classification of Tumors of the Central Nervous System: A summary. Neuro-Oncology.

[3] Stupp R, Taillibert S, Kanner A, Read W, Steinberg D, Lhermitte B (2017). Effect of Tumor-Treating Fields Plus Maintenance Temozolomide vs Maintenance Temozolomide Alone on Survival in Patients With Glioblastoma: A Randomized Clinical Trial. JAMA.

[4] Stupp R, Mason WP, van den Bent MJ, Weller M, Fisher B, Taphoorn MJ (2005). Radiotherapy plus concomitant and adjuvant temozolomide for glioblastoma. New England Journal of Medicine.

[5] Weller M, Cloughesy T, Perry JR, Wick W (2013). Standards of care for treatment of recurrent glioblastoma–are we there yet?. Neuro-Oncology.

[6] Sposto R, Ertel IJ, Jenkin RD, Boesel CP, Venes JL, Ortega JA (1989). The effectiveness of chemotherapy for treatment of high grade astrocytoma in children: results of a randomized trial. A report from the Childrens Cancer Study Group. Journal of Neuro-Oncology.

[7] Cohen KJ, Pollack IF, Zhou T, Buxton A, Holmes EJ, Burger PC (2011). Temozolomide in the treatment of high-grade gliomas in children: A report from the Children's Oncology Group. Neuro-Oncology.

[8] Jakacki RI, Cohen KJ, Buxton A, Krailo MD, Burger PC, Rosenblum MK (2016). Phase 2 study of concurrent radiotherapy and temozolomide followed by temozolomide and lomustine in the treatment of children with high-grade glioma: A report of the Children's Oncology Group ACNS0423 study. Neuro-Oncology.

[9] Cancer Genome Atlas Research N (2008). Comprehensive genomic characterization defines human glioblastoma genes and core pathways. Nature.

[10] Verhaak RG, Hoadley KA, Purdom E, Wang V, Qi Y, Wilkerson MD (2010). Integrated genomic analysis identifies clinically relevant subtypes of glioblastoma characterized by abnormalities in PDGFRA, IDH1, EGFR, and NF1. Cancer Cell.

[11] Brennan CW, Verhaak RG, McKenna A, Campos B, Noushmehr H, Salama SR (2013). The somatic genomic landscape of glioblastoma. Cell.

[12] Sottoriva A, Spiteri I, Piccirillo SG, Touloumis A, Collins VP, Marioni JC (2013). Intratumor heterogeneity in human glioblastoma reflects cancer evolutionary dynamics. Proceedings of the National Academy of Sciences of the United States of America.

[13] Patel AP, Tirosh I, Trombetta JJ, Shalek AK, Gillespie SM, Wakimoto H (2014). Single-cell RNA-seq highlights intratumoral heterogeneity in primary glioblastoma. Science.

[14] Mackay A, Burford A, Carvalho D, Izquierdo E, Fazal-Salom J, Taylor KR (2017). Integrated Molecular Meta-Analysis of 1,000 Pediatric High-Grade and Diffuse Intrinsic Pontine Glioma. Cancer Cell.

[15] Venteicher, A. S., Tirosh, I., Hebert, C., Yizhak, K., Neftel, C., Filbin, M. G., et al. (2017). Decoupling genetics, lineages, and microenvironment in IDH-mutant gliomas by single-cell RNA-seq. *Science, 355*(6332), eaai8478. 10.1126/science.aai8478 10.1126/science.aai8478 PMC5519096 28360267

[16] Vinci M, Burford A, Molinari V, Kessler K, Popov S, Clarke M (2018). Functional diversity and cooperativity between subclonal populations of pediatric glioblastoma and diffuse intrinsic pontine glioma cells. Nature Medicine.

[17] Neftel C, Laffy J, Filbin MG, Hara T, Shore ME, Rahme GJ (2019). An Integrative Model of Cellular States, Plasticity, and Genetics for Glioblastoma. Cell.

[18] Singh SK, Clarke ID, Terasaki M, Bonn VE, Hawkins C, Squire J (2003). Identification of a cancer stem cell in human brain tumors. Cancer Research.

[19] Hemmati HD, Nakano I, Lazareff JA, Masterman-Smith M, Geschwind DH, Bronner-Fraser M (2003). Cancerous stem cells can arise from pediatric brain tumors. Proceedings of the National Academy of Sciences of the United States of America.

[20] Singh SK, Hawkins C, Clarke ID, Squire JA, Bayani J, Hide T (2004). Identification of human brain tumour initiating cells. Nature.

[21] Galli R, Binda E, Orfanelli U, Cipelletti B, Gritti A, De Vitis S (2004). Isolation and characterization of tumorigenic, stem-like neural precursors from human glioblastoma. Cancer Research.

[22] Bao S, Wu Q, McLendon RE, Hao Y, Shi Q, Hjelmeland AB (2006). Glioma stem cells promote radioresistance by preferential activation of the DNA damage response. Nature.

[23] Liu G, Yuan X, Zeng Z, Tunici P, Ng H, Abdulkadir IR (2006). Analysis of gene expression and chemoresistance of CD133+ cancer stem cells in glioblastoma. Molecular Cancer.

[24] Chen J, Li Y, Yu TS, McKay RM, Burns DK, Kernie SG (2012). A restricted cell population propagates glioblastoma growth after chemotherapy. Nature.

[25] Minata M, Audia A, Shi J, Lu S, Bernstock J, Pavlyukov MS (2019). Phenotypic Plasticity of Invasive Edge Glioma Stem-like Cells in Response to Ionizing Radiation. Cell Reports.

[26] Prager BC, Bhargava S, Mahadev V, Hubert CG, Rich JN (2020). Glioblastoma Stem Cells: Driving Resilience through Chaos. Trends in Cancer.

[27] Wong CH, Siah KW, Lo AW (2019). Estimation of clinical trial success rates and related parameters. Biostatistics.

[28] Hambardzumyan D, Bergers G (2015). Glioblastoma: Defining Tumor Niches. Trends Cancer.

[29] Wang Q, Hu B, Hu X, Kim H, Squatrito M, Scarpace L (2017). Tumor Evolution of Glioma-Intrinsic Gene Expression Subtypes Associates with Immunological Changes in the Microenvironment. Cancer Cell.

[30] Broekman ML, Maas SLN, Abels ER, Mempel TR, Krichevsky AM, Breakefield XO (2018). Multidimensional communication in the microenvirons of glioblastoma. Nature Reviews Neurology.

[31] Clement V, Sanchez P, de Tribolet N, Radovanovic I, Ruizi Altaba A (2007). HEDGEHOG-GLI1 Signaling Regulates Human Glioma Growth, Cancer Stem Cell Self-Renewal, and Tumorigenicity. Current Biology.

[32] Venkatesh HS, Johung TB, Caretti V, Noll A, Tang Y, Nagaraja S (2015). Neuronal Activity Promotes Glioma Growth through Neuroligin-3 Secretion. Cell.

[33] Shi Y, Ping Y-F, Zhou W, He Z-C, Chen C, Bian B-S-J (2017). Tumour-associated macrophages secrete pleiotrophin to promote PTPRZ1 signalling in glioblastoma stem cells for tumour growth. Nature Communications.

[34] Pine AR, Cirigliano SM, Nicholson JG, Hu Y, Linkous A, Miyaguchi K (2020). Tumor Microenvironment Is Critical for the Maintenance of Cellular States Found in Primary Glioblastomas. Cancer Discovery.

[35] Neufeld, L., Yeini, E., Reisman, N., Shtilerman, Y., Ben-Shushan, D., Pozzi, S., et al. (2021). Microengineered perfusable 3D-bioprinted glioblastoma model for in vivo mimicry of tumor microenvironment. *Science Advances, 7*(34), eabi9119. 10.1126/sciadv.abi9119 10.1126/sciadv.abi9119 PMC8373143 34407932

[36] Yi H-G, Jeong YH, Kim Y, Choi Y-J, Moon HE, Park SH (2019). A bioprinted human-glioblastoma-on-a-chip for the identification of patient-specific responses to chemoradiotherapy. Nature Biomedical Engineering.

[37] Puget S, Philippe C, Bax DA, Job B, Varlet P, Junier MP (2012). Mesenchymal transition and PDGFRA amplification/mutation are key distinct oncogenic events in pediatric diffuse intrinsic pontine gliomas. PLoS One.

[38] Snuderl M, Fazlollahi L, Le LP, Nitta M, Zhelyazkova BH, Davidson CJ (2011). Mosaic amplification of multiple receptor tyrosine kinase genes in glioblastoma. Cancer Cell.

[39] Filbin MG, Tirosh I, Hovestadt V, Shaw ML, Escalante LE, Mathewson ND (2018). Developmental and oncogenic programs in H3K27M gliomas dissected by single-cell RNA-seq. Science.

[40] Finlay JL, Boyett JM, Yates AJ, Wisoff JH, Milstein JM, Geyer JR (1995). Randomized phase III trial in childhood high-grade astrocytoma comparing vincristine, lomustine, and prednisone with the eight-drugs-in-1-day regimen Childrens Cancer Group. Journal of Clinical Oncology.

[41] Hegi ME, Diserens AC, Godard S, Dietrich PY, Regli L, Ostermann S (2004). Clinical trial substantiates the predictive value of O-6-methylguanine-DNA methyltransferase promoter methylation in glioblastoma patients treated with temozolomide. Clinical Cancer Research.

[42] Hegi ME, Diserens AC, Gorlia T, Hamou MF, de Tribolet N, Weller M (2005). MGMT gene silencing and benefit from temozolomide in glioblastoma. New England Journal of Medicine.

[43] Esteller M, Garcia-Foncillas J, Andion E, Goodman SN, Hidalgo OF, Vanaclocha V (2000). Inactivation of the DNA-repair gene MGMT and the clinical response of gliomas to alkylating agents. New England Journal of Medicine.

[44] Peng L, Fu J, Wang W, Hofman FM, Chen TC, Chen L (2019). Distribution of cancer stem cells in two human brain gliomas. Oncology Letters.

[45] Bastola S, Pavlyukov MS, Yamashita D, Ghosh S, Cho H, Kagaya N (2020). Glioma-initiating cells at tumor edge gain signals from tumor core cells to promote their malignancy. Nature Communications.

[46] Joo KM, Kim SY, Jin X, Song SY, Kong DS, Lee JI (2008). Clinical and biological implications of CD133-positive and CD133-negative cells in glioblastomas. Laboratory Investigation.

[47] Teo WY, Sekar K, Seshachalam P, Shen J, Chow WY, Lau CC (2019). Relevance of a TCGA-derived Glioblastoma Subtype Gene-Classifier among Patient Populations. Science and Reports.

[48] Paugh BS, Qu C, Jones C, Liu Z, Adamowicz-Brice M, Zhang J (2010). Integrated molecular genetic profiling of pediatric high-grade gliomas reveals key differences with the adult disease. Journal of Clinical Oncology.

[49] Sturm D, Witt H, Hovestadt V, Khuong-Quang DA, Jones DT, Konermann C (2012). Hotspot mutations in H3F3A and IDH1 define distinct epigenetic and biological subgroups of glioblastoma. Cancer Cell.

[50] Sturm D, Bender S, Jones DT, Lichter P, Grill J, Becher O (2014). Paediatric and adult glioblastoma: Multiform (epi)genomic culprits emerge. Nature Reviews Cancer.

[51] Jones C, Karajannis MA, Jones DTW, Kieran MW, Monje M, Baker SJ (2017). Pediatric high-grade glioma: Biologically and clinically in need of new thinking. Neuro-Oncology.

[52] Bax DA, Little SE, Gaspar N, Perryman L, Marshall L, Viana-Pereira M (2009). Molecular and phenotypic characterisation of paediatric glioma cell lines as models for preclinical drug development. PLoS One.

[53] Bax DA, Mackay A, Little SE, Carvalho D, Viana-Pereira M, Tamber N (2010). A distinct spectrum of copy number aberrations in pediatric high-grade gliomas. Clinical Cancer Research.

[54] Schwartzentruber J, Korshunov A, Liu XY, Jones DT, Pfaff E, Jacob K (2012). Driver mutations in histone H3.3 and chromatin remodelling genes in paediatric glioblastoma. Nature.

[55] Wu G, Broniscer A, McEachron TA, Lu C, Paugh BS, Becksfort J (2012). Somatic histone H3 alterations in pediatric diffuse intrinsic pontine gliomas and non-brainstem glioblastomas. Nature Genetics.

[56] Korshunov A, Ryzhova M, Hovestadt V, Bender S, Sturm D, Capper D (2015). Integrated analysis of pediatric glioblastoma reveals a subset of biologically favorable tumors with associated molecular prognostic markers. Acta Neuropathologica.

[57] Korshunov A, Schrimpf D, Ryzhova M, Sturm D, Chavez L, Hovestadt V (2017). H3-/IDH-wild type pediatric glioblastoma is comprised of molecularly and prognostically distinct subtypes with associated oncogenic drivers. Acta Neuropathologica.

[58] Stewart LA (2002). Chemotherapy in adult high-grade glioma: A systematic review and meta-analysis of individual patient data from 12 randomised trials. Lancet.

[59] Noushmehr H, Weisenberger DJ, Diefes K, Phillips HS, Pujara K, Berman BP (2010). Identification of a CpG island methylator phenotype that defines a distinct subgroup of glioma. Cancer Cell.

[60] Mur P, Mollejo M, Ruano Y, de Lope ÁR, Fiaño C, García JF (2013). Codeletion of 1p and 19q determines distinct gene methylation and expression profiles in IDH-mutated oligodendroglial tumors. Acta Neuropathologica.

[61] Louis DN, Wesseling P, Aldape K, Brat DJ, Capper D, Cree IA (2020). cIMPACT-NOW update 6: New entity and diagnostic principle recommendations of the cIMPACT-Utrecht meeting on future CNS tumor classification and grading. Brain Pathology.

[62] Louis DN, Perry A, Reifenberger G, von Deimling A, Figarella-Branger D, Cavenee WK (2016). The 2016 World Health Organization Classification of Tumors of the Central Nervous System: A summary. Acta Neuropathologica.

[63] Clarke M, Mackay A, Ismer B, Pickles JC, Tatevossian RG, Newman S (2020). Infant High-Grade Gliomas Comprise Multiple Subgroups Characterized by Novel Targetable Gene Fusions and Favorable Outcomes. Cancer Discovery.

[64] Guerreiro Stucklin AS, Ryall S, Fukuoka K, Zapotocky M, Lassaletta A, Li C (2019). Alterations in ALK/ROS1/NTRK/MET drive a group of infantile hemispheric gliomas. Nature Communications.

[65] Phillips HS, Kharbanda S, Chen R, Forrest WF, Soriano RH, Wu TD (2006). Molecular subclasses of high-grade glioma predict prognosis, delineate a pattern of disease progression, and resemble stages in neurogenesis. Cancer Cell.

[66] Nowell PC (1976). The clonal evolution of tumor cell populations. Science.

[67] Greaves M, Maley CC (2012). Clonal evolution in cancer. Nature.

[68] Dagogo-Jack I, Shaw AT (2018). Tumour heterogeneity and resistance to cancer therapies. Nature Reviews Clinical Oncology.

[69] Wang J, Cazzato E, Ladewig E, Frattini V, Rosenbloom DI, Zairis S (2016). Clonal evolution of glioblastoma under therapy. Nature Genetics.

[70] Meyer M, Reimand J, Lan X, Head R, Zhu X, Kushida M (2015). Single cell-derived clonal analysis of human glioblastoma links functional and genomic heterogeneity. Proceedings of the National Academy of Sciences of the United States of America.

[71] Bhang HE, Ruddy DA, Krishnamurthy Radhakrishna V, Caushi JX, Zhao R, Hims MM (2015). Studying clonal dynamics in response to cancer therapy using high-complexity barcoding. Nature Medicine.

[72] Hoffman M, Gillmor AH, Kunz DJ, Johnston MJ, Nikolic A, Narta K (2019). Intratumoral Genetic and Functional Heterogeneity in Pediatric Glioblastoma. Cancer Research.

[73] Greaves M (2013). Cancer stem cells as 'units of selection'. Evolutionary Applications.

[74] Kreso A, Dick JE (2014). Evolution of the cancer stem cell model. Cell Stem Cell.

[75] Shackleton M, Quintana E, Fearon ER, Morrison SJ (2009). Heterogeneity in cancer: Cancer stem cells versus clonal evolution. Cell.

[76] Piccirillo SG, Combi R, Cajola L, Patrizi A, Redaelli S, Bentivegna A (2009). Distinct pools of cancer stem-like cells coexist within human glioblastomas and display different tumorigenicity and independent genomic evolution. Oncogene.

[77] Couturier CP, Ayyadhury S, Le PU, Nadaf J, Monlong J, Riva G (2020). Single-cell RNA-seq reveals that glioblastoma recapitulates a normal neurodevelopmental hierarchy. Nature Communications.

[78] Tirosh I, Venteicher AS, Hebert C, Escalante LE, Patel AP, Yizhak K (2016). Single-cell RNA-seq supports a developmental hierarchy in human oligodendroglioma. Nature.

[79] Sun Y, Goderie SK, Temple S (2005). Asymmetric distribution of EGFR receptor during mitosis generates diverse CNS progenitor cells. Neuron.

[80] Lim S, Kaldis P (2012). Loss of Cdk2 and Cdk4 induces a switch from proliferation to differentiation in neural stem cells. Stem Cells.

[81] Zhu Q, Zhao X, Zheng K, Li H, Huang H, Zhang Z (2014). Genetic evidence that Nkx2.2 and Pdgfra are major determinants of the timing of oligodendrocyte differentiation in the developing CNS. Development.

[82] Suva ML, Tirosh I (2020). The Glioma Stem Cell Model in the Era of Single-Cell Genomics. Cancer Cell.

[83] Chaligne R, Gaiti F, Silverbush D, Schiffman JS, Weisman HR, Kluegel L (2021). Epigenetic encoding, heritability and plasticity of glioma transcriptional cell states. Nature Genetics.

[84] Lan X, Jorg DJ, Cavalli FMG, Richards LM, Nguyen LV, Vanner RJ (2017). Fate mapping of human glioblastoma reveals an invariant stem cell hierarchy. Nature.

[85] Bhaduri A, Di Lullo E, Jung D, Muller S, Crouch EE, Espinosa CS (2020). Outer Radial Glia-like Cancer Stem Cells Contribute to Heterogeneity of Glioblastoma. Cell Stem Cell.

[86] Liau BB, Sievers C, Donohue LK, Gillespie SM, Flavahan WA, Miller TE (2017). Adaptive Chromatin Remodeling Drives Glioblastoma Stem Cell Plasticity and Drug Tolerance. Cell Stem Cell.

[87] Vitale I, Manic G, De Maria R, Kroemer G, Galluzzi L (2017). DNA Damage in Stem Cells. Molecular Cell.

[88] Cheng L, Wu Q, Huang Z, Guryanova OA, Huang Q, Shou W (2011). L1CAM regulates DNA damage checkpoint response of glioblastoma stem cells through NBS1. EMBO Journal.

[89] De Bacco F, D'Ambrosio A, Casanova E, Orzan F, Neggia R, Albano R (2016). MET inhibition overcomes radiation resistance of glioblastoma stem-like cells. EMBO Molecular Medicine.

[90] King HO, Brend T, Payne HL, Wright A, Ward TA, Patel K (2017). RAD51 Is a Selective DNA Repair Target to Radiosensitize Glioma Stem Cells. Stem Cell Reports.

[91] Ahmed SU, Carruthers R, Gilmour L, Yildirim S, Watts C, Chalmers AJ (2015). Selective Inhibition of Parallel DNA Damage Response Pathways Optimizes Radiosensitization of Glioblastoma Stem-like Cells. Cancer Research.

[92] Carruthers R, Ahmed SU, Strathdee K, Gomez-Roman N, Amoah-Buahin E, Watts C (2015). Abrogation of radioresistance in glioblastoma stem-like cells by inhibition of ATM kinase. Molecular Oncology.

[93] Tamura K, Aoyagi M, Wakimoto H, Ando N, Nariai T, Yamamoto M (2010). Accumulation of CD133-positive glioma cells after high-dose irradiation by Gamma Knife surgery plus external beam radiation. Journal of Neurosurgery.

[94] Lee J, Kotliarova S, Kotliarov Y, Li A, Su Q, Donin NM (2006). Tumor stem cells derived from glioblastomas cultured in bFGF and EGF more closely mirror the phenotype and genotype of primary tumors than do serum-cultured cell lines. Cancer Cell.

[95] Jin X, Kim LJY, Wu Q, Wallace LC, Prager BC, Sanvoranart T (2017). Targeting glioma stem cells through combined BMI1 and EZH2 inhibition. Nature Medicine.

[96] Prabhu A, Kesarwani P, Kant S, Graham SF, Chinnaiyan P (2017). Histologically defined intratumoral sequencing uncovers evolutionary cues into conserved molecular events driving gliomagenesis. Neuro-Oncology.

[97] Darmanis S, Sloan SA, Croote D, Mignardi M, Chernikova S, Samghababi P (2017). Single-Cell RNA-Seq Analysis of Infiltrating Neoplastic Cells at the Migrating Front of Human Glioblastoma. Cell Reports.

[98] Zagzag D, Esencay M, Mendez O, Yee H, Smirnova I, Huang Y (2008). Hypoxia- and vascular endothelial growth factor-induced stromal cell-derived factor-1alpha/CXCR4 expression in glioblastomas: One plausible explanation of Scherer's structures. American Journal of Pathology.

[99] Yadav VN, Zamler D, Baker GJ, Kadiyala P, Erdreich-Epstein A, DeCarvalho AC (2016). CXCR4 increases in-vivo glioma perivascular invasion, and reduces radiation induced apoptosis: A genetic knockdown study. Oncotarget.

[100] Gravina GL, Mancini A, Colapietro A, Vitale F, Vetuschi A, Pompili S (2017). The novel CXCR4 antagonist, PRX177561, reduces tumor cell proliferation and accelerates cancer stem cell differentiation in glioblastoma preclinical models. Tumour Biology.

[101] Montana V, Sontheimer H (2011). Bradykinin promotes the chemotactic invasion of primary brain tumors. Journal of Neuroscience.

[102] Seifert S, Sontheimer H (2014). Bradykinin enhances invasion of malignant glioma into the brain parenchyma by inducing cells to undergo amoeboid migration. Journal of Physiology.

[103] Fan X, Khaki L, Zhu TS, Soules ME, Talsma CE, Gul N (2010). NOTCH pathway blockade depletes CD133-positive glioblastoma cells and inhibits growth of tumor neurospheres and xenografts. Stem Cells.

[104] Charles N, Ozawa T, Squatrito M, Bleau AM, Brennan CW, Hambardzumyan D (2010). Perivascular nitric oxide activates notch signaling and promotes stem-like character in PDGF-induced glioma cells. Cell Stem Cell.

[105] Hovinga KE, Shimizu F, Wang R, Panagiotakos G, Van Der Heijden M, Moayedpardazi H (2010). Inhibition of notch signaling in glioblastoma targets cancer stem cells via an endothelial cell intermediate. Stem Cells.

[106] Zhu TS, Costello MA, Talsma CE, Flack CG, Crowley JG, Hamm LL (2011). Endothelial cells create a stem cell niche in glioblastoma by providing NOTCH ligands that nurture self-renewal of cancer stem-like cells. Cancer Research.

[107] Jeon HM, Kim SH, Jin X, Park JB, Kim SH, Joshi K (2014). Crosstalk between glioma-initiating cells and endothelial cells drives tumor progression. Cancer Research.

[108] Clara JA, Monge C, Yang Y, Takebe N (2020). Targeting signalling pathways and the immune microenvironment of cancer stem cells - a clinical update. Nature Reviews Clinical Oncology.

[109] McCoy MG, Nyanyo D, Hung CK, Goerger JP, Zipfel WR, Williams RM (2019). Endothelial cells promote 3D invasion of GBM by IL-8-dependent induction of cancer stem cell properties. Sci Rep.

[110] Li D, Tian Y, Hu Y, Qi Y, Tian N, Li S (2019). Glioma-associated human endothelial cell-derived extracellular vesicles specifically promote the tumourigenicity of glioma stem cells via CD9. Oncogene.

[111] Semenza GL (2013). HIF-1 mediates metabolic responses to intratumoral hypoxia and oncogenic mutations. The Journal of Clinical Investigation.

[112] Ishii A, Kimura T, Sadahiro H, Kawano H, Takubo K, Suzuki M (2016). Histological Characterization of the Tumorigenic "Peri-Necrotic Niche" Harboring Quiescent Stem-Like Tumor Cells in Glioblastoma. PLoS One.

[113] Dirkse A, Golebiewska A, Buder T, Nazarov PV, Muller A, Poovathingal S (2019). Stem cell-associated heterogeneity in Glioblastoma results from intrinsic tumor plasticity shaped by the microenvironment. Nature Communications.

[114] Wang D, Berglund AE, Kenchappa RS, MacAulay RJ, Mulé JJ, Etame AB (2017). BIRC3 is a biomarker of mesenchymal habitat of glioblastoma, and a mediator of survival adaptation in hypoxia-driven glioblastoma habitats. Scientific Reports.

[115] Liu J, Gao L, Zhan N, Xu P, Yang J, Yuan F (2020). Hypoxia induced ferritin light chain (FTL) promoted epithelia mesenchymal transition and chemoresistance of glioma. Journal of Experimental & Clinical Cancer Research.

[116] Azzam EI, Jay-Gerin JP, Pain D (2012). Ionizing radiation-induced metabolic oxidative stress and prolonged cell injury. Cancer Letters.

[117] Perillo B, Di Donato M, Pezone A, Di Zazzo E, Giovannelli P, Galasso G (2020). ROS in cancer therapy: The bright side of the moon. Experimental & Molecular Medicine.

[118] Matschke J, Riffkin H, Klein D, Handrick R, Ludemann L, Metzen E (2016). Targeted Inhibition of Glutamine-Dependent Glutathione Metabolism Overcomes Death Resistance Induced by Chronic Cycling Hypoxia. Antioxidants & Redox Signaling.

[119] Schaich M, Kestel L, Pfirrmann M, Robel K, Illmer T, Kramer M (2009). A MDR1 (ABCB1) gene single nucleotide polymorphism predicts outcome of temozolomide treatment in glioblastoma patients. Annals of Oncology.

[120] Chou CW, Wang CC, Wu CP, Lin YJ, Lee YC, Cheng YW (2012). Tumor cycling hypoxia induces chemoresistance in glioblastoma multiforme by upregulating the expression and function of ABCB1. Neuro-Oncology.

[121] Munoz JL, Walker ND, Scotto KW, Rameshwar P (2015). Temozolomide competes for P-glycoprotein and contributes to chemoresistance in glioblastoma cells. Cancer Letters.

[122] Soeda A, Park M, Lee D, Mintz A, Androutsellis-Theotokis A, McKay RD (2009). Hypoxia promotes expansion of the CD133-positive glioma stem cells through activation of HIF-1α. Oncogene.

[123] Simons M, Gordon E, Claesson-Welsh L (2016). Mechanisms and regulation of endothelial VEGF receptor signalling. Nature Reviews Molecular Cell Biology.

[124] Zagzag D, Lukyanov Y, Lan L, Ali MA, Esencay M, Mendez O (2006). Hypoxia-inducible factor 1 and VEGF upregulate CXCR4 in glioblastoma: Implications for angiogenesis and glioma cell invasion. Laboratory Investigation.

[125] Dong J, Zhao Y, Huang Q, Fei X, Diao Y, Shen Y (2011). Glioma stem/progenitor cells contribute to neovascularization via transdifferentiation. Stem Cell Rev Rep.

[126] Soda Y, Marumoto T, Friedmann-Morvinski D, Soda M, Liu F, Michiue H (2011). Transdifferentiation of glioblastoma cells into vascular endothelial cells. Proceedings of the National Academy of Sciences of the United States of America.

[127] Wang R, Chadalavada K, Wilshire J, Kowalik U, Hovinga KE, Geber A (2010). Glioblastoma stem-like cells give rise to tumour endothelium. Nature.

[128] Mao XG, Xue XY, Wang L, Zhang X, Yan M, Tu YY (2013). CDH5 is specifically activated in glioblastoma stemlike cells and contributes to vasculogenic mimicry induced by hypoxia. Neuro-Oncology.

[129] Wang J, Xu S-L, Duan J-J, Yi L, Guo Y-F, Shi Y (2019). Invasion of white matter tracts by glioma stem cells is regulated by a NOTCH1–SOX2 positive-feedback loop. Nature Neuroscience.

[130] Zhang S, Xiong X, Sun Y (2020). Functional characterization of SOX2 as an anticancer target. Signal Transduction and Targeted Therapy.

[131] Venkatesh HS, Tam LT, Woo PJ, Lennon J, Nagaraja S, Gillespie SM (2017). Targeting neuronal activity-regulated neuroligin-3 dependency in high-grade glioma. Nature.

[132] Venkatesh HS, Morishita W, Geraghty AC, Silverbush D, Gillespie SM, Arzt M (2019). Electrical and synaptic integration of glioma into neural circuits. Nature.

[133] Venkataramani V, Tanev DI, Strahle C, Studier-Fischer A, Fankhauser L, Kessler T (2019). Glutamatergic synaptic input to glioma cells drives brain tumour progression. Nature.

[134] Yu-Ju Wu C, Chen CH, Lin CY, Feng LY, Lin YC, Wei KC (2020). CCL5 of glioma-associated microglia/macrophages regulates glioma migration and invasion via calcium-dependent matrix metalloproteinase 2. Neuro-Oncology.

[135] Hambardzumyan D, Gutmann DH, Kettenmann H (2016). The role of microglia and macrophages in glioma maintenance and progression. Nature Neuroscience.

[136] Vinnakota K, Hu F, Ku MC, Georgieva PB, Szulzewsky F, Pohlmann A (2013). Toll-like receptor 2 mediates microglia/brain macrophage MT1-MMP expression and glioma expansion. Neuro-Oncology.

[137] Ye XZ, Xu SL, Xin YH, Yu SC, Ping YF, Chen L (2012). Tumor-associated microglia/macrophages enhance the invasion of glioma stem-like cells via TGF-beta1 signaling pathway. The Journal of Immunology.

[138] Baker GJ, Yadav VN, Motsch S, Koschmann C, Calinescu AA, Mineharu Y (2014). Mechanisms of glioma formation: Iterative perivascular glioma growth and invasion leads to tumor progression, VEGF-independent vascularization, and resistance to antiangiogenic therapy. Neoplasia.

[139] Liu CJ, Shamsan GA, Akkin T, Odde DJ (2019). Glioma Cell Migration Dynamics in Brain Tissue Assessed by Multimodal Optical Imaging. Biophysical Journal.

[140] Goffart N, Kroonen J, Di Valentin E, Dedobbeleer M, Denne A, Martinive P (2015). Adult mouse subventricular zones stimulate glioblastoma stem cells specific invasion through CXCL12/CXCR4 signaling. Neuro-Oncology.

[141] Favaro R, Valotta M, Ferri ALM, Latorre E, Mariani J, Giachino C (2009). Hippocampal development and neural stem cell maintenance require Sox2-dependent regulation of Shh. Nature Neuroscience.

[142] Goldman SA, Chen Z (2011). Perivascular instruction of cell genesis and fate in the adult brain. Nature Neuroscience.

[143] Shen Q, Goderie SK, Jin L, Karanth N, Sun Y, Abramova N (2004). Endothelial cells stimulate self-renewal and expand neurogenesis of neural stem cells. Science.

[144] Palmer TD, Willhoite AR, Gage FH (2000). Vascular niche for adult hippocampal neurogenesis. The Journal of Comparative Neurology.

[145] Kazanis I, Lathia JD, Vadakkan TJ, Raborn E, Wan R, Mughal MR (2010). Quiescence and activation of stem and precursor cell populations in the subependymal zone of the mammalian brain are associated with distinct cellular and extracellular matrix signals. Journal of Neuroscience.

[146] Yan GN, Yang L, Lv YF, Shi Y, Shen LL, Yao XH (2014). Endothelial cells promote stem-like phenotype of glioma cells through activating the Hedgehog pathway. The Journal of Pathology.

[147] Majmundar AJ, Wong WJ, Simon MC (2010). Hypoxia-inducible factors and the response to hypoxic stress. Molecular Cell.

[148] Forristal CE, Wright KL, Hanley NA, Oreffo RO, Houghton FD (2010). Hypoxia inducible factors regulate pluripotency and proliferation in human embryonic stem cells cultured at reduced oxygen tensions. Reproduction.

[149] Petruzzelli R, Christensen DR, Parry KL, Sanchez-Elsner T, Houghton FD (2014). HIF-2alpha regulates NANOG expression in human embryonic stem cells following hypoxia and reoxygenation through the interaction with an Oct-Sox cis regulatory element. PLoS One.

[150] Arthur SA, Blaydes JP, Houghton FD (2019). Glycolysis Regulates Human Embryonic Stem Cell Self-Renewal under Hypoxia through HIF-2alpha and the Glycolytic Sensors CTBPs. Stem Cell Reports.

[151] Li Z, Bao S, Wu Q, Wang H, Eyler C, Sathornsumetee S (2009). Hypoxia-inducible factors regulate tumorigenic capacity of glioma stem cells. Cancer Cell.

[152] Heddleston JM, Li Z, McLendon RE, Hjelmeland AB, Rich JN (2009). The hypoxic microenvironment maintains glioblastoma stem cells and promotes reprogramming towards a cancer stem cell phenotype. Cell Cycle.

[153] Aulestia FJ, Neant I, Dong J, Haiech J, Kilhoffer MC, Moreau M (2018). Quiescence status of glioblastoma stem-like cells involves remodelling of Ca(2+) signalling and mitochondrial shape. Science and Reports.

[154] Dong J, Aulestia FJ, Assad Kahn S, Zeniou M, Dubois LG, El-Habr EA (2017). Bisacodyl and its cytotoxic activity on human glioblastoma stem-like cells. Implication of inositol 1,4,5-triphosphate receptor dependent calcium signaling. Biochimica et Biophysica Acta (BBA) - Molecular Cell Research.

[155] Tejero R, Huang Y, Katsyv I, Kluge M, Lin JY, Tome-Garcia J (2019). Gene signatures of quiescent glioblastoma cells reveal mesenchymal shift and interactions with niche microenvironment. EBioMedicine.

[156] Wang D, Berglund A, Kenchappa RS, Forsyth PA, Mulé JJ, Etame AB (2016). BIRC3 is a novel driver of therapeutic resistance in Glioblastoma. Scientific Reports.

[157] Cuddapah VA, Robel S, Watkins S, Sontheimer H (2014). A neurocentric perspective on glioma invasion. Nature Reviews Neuroscience.

[158] Esmaeili M, Stensjoen AL, Berntsen EM, Solheim O, Reinertsen I (2018). The Direction of Tumour Growth in Glioblastoma Patients. Science and Reports.

[159] Watkins S, Robel S, Kimbrough IF, Robert SM, Ellis-Davies G, Sontheimer H (2014). Disruption of astrocyte-vascular coupling and the blood-brain barrier by invading glioma cells. Nature Communications.

[160] Osswald M, Jung E, Sahm F, Solecki G, Venkataramani V, Blaes J (2015). Brain tumour cells interconnect to a functional and resistant network. Nature.

[161] Tombal B, Denmeade SR, Gillis JM, Isaacs JT (2002). A supramicromolar elevation of intracellular free calcium ([Ca2+]i) is consistently required to induce the execution phase of apoptosis. Cell Death & Differentiation.

[162] Weil S, Osswald M, Solecki G, Grosch J, Jung E, Lemke D (2017). Tumor microtubes convey resistance to surgical lesions and chemotherapy in gliomas. Neuro-Oncology.

[163] Südhof TC (2008). Neuroligins and neurexins link synaptic function to cognitive disease. Nature.

[164] Varoqueaux F, Aramuni G, Rawson RL, Mohrmann R, Missler M, Gottmann K (2006). Neuroligins determine synapse maturation and function. Neuron.

[165] Liu R, Qin XP, Zhuang Y, Zhang Y, Liao HB, Tang JC (2018). Glioblastoma recurrence correlates with NLGN3 levels. Cancer Medicine.

[166] Derks J, Wesseling P, Carbo EWS, Hillebrand A, van Dellen E, de Witt Hamer PC (2018). Oscillatory brain activity associates with neuroligin-3 expression and predicts progression free survival in patients with diffuse glioma. Journal of Neuro-Oncology.

[167] Belgers V, Numan T, Kulik SD, Hillebrand A, de Witt Hamer PC, Geurts JJG (2020). Postoperative oscillatory brain activity as an add-on prognostic marker in diffuse glioma. Journal of Neuro-oncology.

[168] Qu M, Qiu BO, Xiong W, Chen D, Wu A (2015). Expression of a-disintegrin and metalloproteinase 10 correlates with grade of malignancy in human glioma. Oncology Letters.

[169] Bergles DE, Roberts JDB, Somogyi P, Jahr CE (2000). Glutamatergic synapses on oligodendrocyte precursor cells in the hippocampus. Nature.

[170] Mackay A, Burford A, Molinari V, Jones DTW, Izquierdo E, Brouwer-Visser J (2018). Molecular, Pathological, Radiological, and Immune Profiling of Non-brainstem Pediatric High-Grade Glioma from the HERBY Phase II Randomized Trial. Cancer Cell.

[171] Ross JL, Chen Z, Herting CJ, Grabovska Y, Szulzewsky F, Puigdelloses M (2021). Platelet-derived growth factor beta is a potent inflammatory driver in paediatric high-grade glioma. Brain.

[172] Chen Z, Feng X, Herting CJ, Garcia VA, Nie K, Pong WW (2017). Cellular and Molecular Identity of Tumor-Associated Macrophages in Glioblastoma. Cancer Research.

[173] Bettinger I, Thanos S, Paulus W (2002). Microglia promote glioma migration. Acta Neuropathologica.

[174] Winkler J, Abisoye-Ogunniyan A, Metcalf KJ, Werb Z (2020). Concepts of extracellular matrix remodelling in tumour progression and metastasis. Nature Communications.

[175] Meng Y, Beckett MA, Liang H, Mauceri HJ, van Rooijen N, Cohen KS (2010). Blockade of tumor necrosis factor alpha signaling in tumor-associated macrophages as a radiosensitizing strategy. Cancer Research.

[176] Yi L, Xiao H, Xu M, Ye X, Hu J, Li F (2011). Glioma-initiating cells: A predominant role in microglia/macrophages tropism to glioma. Journal of Neuroimmunology.

[177] Guo X, Xue H, Shao Q, Wang J, Guo X, Chen X (2016). Hypoxia promotes glioma-associated macrophage infiltration via periostin and subsequent M2 polarization by upregulating TGF-beta and M-CSFR. Oncotarget.

[178] Zhou W, Ke SQ, Huang Z, Flavahan W, Fang X, Paul J (2015). Periostin secreted by glioblastoma stem cells recruits M2 tumour-associated macrophages and promotes malignant growth. Nature Cell Biology.

[179] Ben-David U, Ha G, Tseng YY, Greenwald NF, Oh C, Shih J (2017). Patient-derived xenografts undergo mouse-specific tumor evolution. Nature Genetics.

[180] Ben-David U, Siranosian B, Ha G, Tang H, Oren Y, Hinohara K (2018). Genetic and transcriptional evolution alters cancer cell line drug response. Nature.

[181] Hutchinson L, Kirk R (2011). High drug attrition rates–where are we going wrong?. Nature Reviews Clinical Oncology.

[182] Herculano-Houzel S (2014). The glia/neuron ratio: How it varies uniformly across brain structures and species and what that means for brain physiology and evolution. Glia.

[183] Roper J, Hung KE (2012). Priceless GEMMs: Genetically engineered mouse models for colorectal cancer drug development. Trends in Pharmacological Sciences.

[184] Gillet JP, Varma S, Gottesman MM (2013). The clinical relevance of cancer cell lines. Journal of the National Cancer Institute.

[185] Pamies D, Zurich MG, Hartung T (2020). Organotypic Models to Study Human Glioblastoma: Studying the Beast in Its Ecosystem. iScience.

[186] Reinartz R, Wang S, Kebir S, Silver DJ, Wieland A, Zheng T (2017). Functional Subclone Profiling for Prediction of Treatment-Induced Intratumor Population Shifts and Discovery of Rational Drug Combinations in Human Glioblastoma. Clinical Cancer Research.

[187] Lathia JD, Mack SC, Mulkearns-Hubert EE, Valentim CL, Rich JN (2015). Cancer stem cells in glioblastoma. Genes & Development.

[188] Hubert CG, Rivera M, Spangler LC, Wu Q, Mack SC, Prager BC (2016). A Three-Dimensional Organoid Culture System Derived from Human Glioblastomas Recapitulates the Hypoxic Gradients and Cancer Stem Cell Heterogeneity of Tumors Found In Vivo. Cancer Research.

[189] Jacob F, Salinas RD, Zhang DY, Nguyen PTT, Schnoll JG, Wong SZH (2020). A Patient-Derived Glioblastoma Organoid Model and Biobank Recapitulates Inter- and Intra-tumoral Heterogeneity. Cell.

[190] US National Library of Medicine (2020). ClinicalTrials.gov NCT04295759. https://clinicaltrials.gov/ct2/show/NCT04295759. Accessed 24 June 2022

[191] Makalowski W, Zhang J, Boguski MS (1996). Comparative analysis of 1196 orthologous mouse and human full-length mRNA and protein sequences. Genome Research.

[192] Sharpless NE, DePinho RA (2006). The mighty mouse: Genetically engineered mouse models in cancer drug development. Nature Reviews Drug Discovery.

[193] Chow LM, Endersby R, Zhu X, Rankin S, Qu C, Zhang J (2011). Cooperativity within and among Pten, p53, and Rb pathways induces high-grade astrocytoma in adult brain. Cancer Cell.

[194] Miller TE, Liau BB, Wallace LC, Morton AR, Xie Q, Dixit D (2017). Transcription elongation factors represent in vivo cancer dependencies in glioblastoma. Nature.

[195] Wei X, Meel MH, Breur M, Bugiani M, Hulleman E, Phoenix TN (2021). Defining tumor-associated vascular heterogeneity in pediatric high-grade and diffuse midline gliomas. Acta Neuropathologica Communications.

[196] He C, Xu K, Zhu X, Dunphy PS, Gudenas B, Lin W (2021). Patient-derived models recapitulate heterogeneity of molecular signatures and drug response in pediatric high-grade glioma. Nature Communications.

[197] Cassidy JW, Caldas C, Bruna A (2015). Maintaining Tumor Heterogeneity in Patient-Derived Tumor Xenografts. Cancer Research.

[198] Thomson JA, Itskovitz-Eldor J, Shapiro SS, Waknitz MA, Swiergiel JJ, Marshall VS (1998). Embryonic Stem Cell Lines Derived from Human Blastocysts. Science.

[199] Takahashi K, Tanabe K, Ohnuki M, Narita M, Ichisaka T, Tomoda K (2007). Induction of Pluripotent Stem Cells from Adult Human Fibroblasts by Defined Factors. Cell.

[200] Takahashi K, Yamanaka S (2006). Induction of Pluripotent Stem Cells from Mouse Embryonic and Adult Fibroblast Cultures by Defined Factors. Cell.

[201] Lancaster MA, Renner M, Martin CA, Wenzel D, Bicknell LS, Hurles ME (2013). Cerebral organoids model human brain development and microcephaly. Nature.

[202] Linkous A, Balamatsias D, Snuderl M, Edwards L, Miyaguchi K, Milner T (2019). Modeling Patient-Derived Glioblastoma with Cerebral Organoids. Cell Reports.

[203] Lancaster MA, Knoblich JA (2014). Generation of cerebral organoids from human pluripotent stem cells. Nature Protocols.

[204] Bian S, Repic M, Guo Z, Kavirayani A, Burkard T, Bagley JA (2018). Genetically engineered cerebral organoids model brain tumor formation. Nature Methods.

[205] Ogawa J, Pao GM, Shokhirev MN, Verma IM (2018). Glioblastoma Model Using Human Cerebral Organoids. Cell Reports.

[206] Krieger TG, Tirier SM, Park J, Jechow K, Eisemann T, Peterziel H (2020). Modeling glioblastoma invasion using human brain organoids and single-cell transcriptomics. Neuro-Oncology.

[207] Goranci-Buzhala G, Mariappan A, Gabriel E, Ramani A, Ricci-Vitiani L, Buccarelli M (2020). Rapid and Efficient Invasion Assay of Glioblastoma in Human Brain Organoids. Cell Reports.

[208] Gordon A, Yoon S-J, Tran SS, Makinson CD, Park JY, Andersen J (2021). Long-term maturation of human cortical organoids matches key early postnatal transitions. Nature Neuroscience.

[209] Warren D, Tomaskovic-Crook E, Wallace GG, Crook JM (2021). Engineering in vitro human neural tissue analogs by 3D bioprinting and electrostimulation. APL Bioeng.

[210] Tang M, Xie Q, Gimple RC, Zhong Z, Tam T, Tian J (2020). Three-dimensional bioprinted glioblastoma microenvironments model cellular dependencies and immune interactions. Cell Research.

[211] Dai X, Ma C, Lan Q, Xu T (2016). 3D bioprinted glioma stem cells for brain tumor model and applications of drug susceptibility. Biofabrication.

[212] Wang X, Li X, Zhang Y, Long X, Zhang H, Xu T (2021). Coaxially Bioprinted Cell-Laden Tubular-Like Structure for Studying Glioma Angiogenesis. Frontiers in bioengineering and biotechnology.

[213] Trujillo-de Santiago, G., Flores-Garza, B. G., Tavares-Negrete, J. A., Lara-Mayorga, I. M., Gonzalez-Gamboa, I., Zhang, Y. S., et al. (2019). The tumor-on-chip: Recent advances in the development of microfluidic systems to recapitulate the physiology of solid tumors. *Materials (Basel), 12*(18), 2945. 10.3390/ma12182945 10.3390/ma12182945 PMC6766252 31514390

[214] Liu X, Fang J, Huang S, Wu X, Xie X, Wang J (2021). Tumor-on-a-chip: From bioinspired design to biomedical application. Microsystems & Nanoengineering.

[215] Petreus T, Cadogan E, Hughes G, Smith A, Pilla Reddy V, Lau A (2021). Tumour-on-chip microfluidic platform for assessment of drug pharmacokinetics and treatment response. Communications Biology.

[216] Silvani G, Basirun C, Wu H, Mehner C, Poole K, Bradbury P (2021). A 3D-Bioprinted Vascularized Glioblastoma-on-a-Chip for Studying the Impact of Simulated Microgravity as a Novel Pre-Clinical Approach in Brain Tumor Therapy. Advanced Therapeutics.

[217] Schmidt S, Alt Y, Deoghare N, Krüger S, Kern A, Rockel AF (2022). A Blood Vessel Organoid Model Recapitulating Aspects of Vasculogenesis, Angiogenesis and Vessel Wall Maturation. Organoids.

[218] Truong D, Fiorelli R, Barrientos ES, Melendez EL, Sanai N, Mehta S (2019). A three-dimensional (3D) organotypic microfluidic model for glioma stem cells - Vascular interactions. Biomaterials.

[219] Straehla JP, Hajal C, Safford HC, Offeddu GS, Boehnke N, Dacoba TG (2022). A predictive microfluidic model of human glioblastoma to assess trafficking of blood-brain barrier-penetrant nanoparticles. Proceedings of the National Academy of Sciences of the United States of America.

[220] Demeule M, Currie JC, Bertrand Y, Che C, Nguyen T, Regina A (2008). Involvement of the low-density lipoprotein receptor-related protein in the transcytosis of the brain delivery vector angiopep-2. Journal of Neurochemistry.

[221] Xiao Y, Kim D, Dura B, Zhang K, Yan R, Li H (2019). Ex vivo Dynamics of Human Glioblastoma Cells in a Microvasculature-on-a-Chip System Correlates with Tumor Heterogeneity and Subtypes. Advanced Science.

[222] Halldorsson S, Lucumi E, Gómez-Sjöberg R, Fleming RMT (2015). Advantages and challenges of microfluidic cell culture in polydimethylsiloxane devices. Biosensors and Bioelectronics.

[223] Balint R, Cassidy NJ, Cartmell SH (2013). Electrical stimulation: A novel tool for tissue engineering. Tissue Engineering Part B Reviews.

[224] Stewart E, Kobayashi NR, Higgins MJ, Quigley AF, Jamali S, Moulton SE (2015). Electrical stimulation using conductive polymer polypyrrole promotes differentiation of human neural stem cells: A biocompatible platform for translational neural tissue engineering. Tissue Engineering Part C Methods.

[225] Tomaskovic-Crook E, Zhang P, Ahtiainen A, Kaisvuo H, Lee CY, Beirne S (2019). Human Neural Tissues from Neural Stem Cells Using Conductive Biogel and Printed Polymer Microelectrode Arrays for 3D Electrical Stimulation. Advanced Healthcare Materials.

[226] Tomaskovic-Crook, E., Higginbottom, S. L., James, E. C., Rathbone, S. J. C., & Crook, J. M. (2020). Electroceuticals for neural regenerative nanomedicine. In M. Razavi (Ed.), *Neural Regenerative Medicine*: Elsevier

[227] Tomaskovic-Crook, E., Gu, Q., Rahim, S. N. A., Wallace, G. G., & Crook, J. M. (2020). Conducting polymer mediated electrical stimulation induces multilineage differentiation with robust neuronal fate determination of human induced pluripotent stem cells. *Cells, 9*(3), 658. 10.3390/cells9030658 10.3390/cells9030658 PMC7140718 32182797

[228] Sauer H, Bekhite MM, Hescheler J, Wartenberg M (2005). Redox control of angiogenic factors and CD31-positive vessel-like structures in mouse embryonic stem cells after direct current electrical field stimulation. Experimental Cell Research.

[229] Geng K, Wang J, Liu P, Tian X, Liu H, Wang X (2019). Electrical stimulation facilitates the angiogenesis of human umbilical vein endothelial cells through MAPK/ERK signaling pathway by stimulating FGF2 secretion. American Journal of Physiology Cell Physiology.

[230] Zhao M, Bai H, Wang E, Forrester JV, McCaig CD (2004). Electrical stimulation directly induces pre-angiogenic responses in vascular endothelial cells by signaling through VEGF receptors. Journal of Cell Science.

[231] Di Lullo E, Kriegstein AR (2017). The use of brain organoids to investigate neural development and disease. Nature Reviews Neuroscience.

[232] Kleinman HK, McGarvey ML, Hassell JR, Star VL, Cannon FB, Laurie GW (1986). Basement membrane complexes with biological activity. Biochemistry.

[233] Kleinman HK, McGarvey ML, Liotta LA, Robey PG, Tryggvason K, Martin GR (1982). Isolation and characterization of type IV procollagen, laminin, and heparan sulfate proteoglycan from the EHS sarcoma. Biochemistry.

[234] Hughes CS, Postovit LM, Lajoie GA (2010). Matrigel: A complex protein mixture required for optimal growth of cell culture. Proteomics.

[235] Kozlowski MT, Crook CJ, Ku HT (2021). Towards organoid culture without Matrigel. Communications Biology.

[236] Tomaskovic-Crook E, Crook JM (2019). Clinically Amendable, Defined, and Rapid Induction of Human Brain Organoids from Induced Pluripotent Stem Cells. Methods in Molecular Biology.

[237] Tomaskovic-Crook E, Higginbottom SL, Zhang B, Bourke J, Wallace GG, Crook JM (2023). Defined, Simplified, Scalable, and Clinically Compatible Hydrogel-Based Production of Human Brain Organoids. Organoids.

[238] Shirahama H, Lee BH, Tan LP, Cho NJ (2016). Precise Tuning of Facile One-Pot Gelatin Methacryloyl (GelMA) Synthesis. Science and Reports.

[239] Van Den Bulcke AI, Bogdanov B, De Rooze N, Schacht EH, Cornelissen M, Berghmans H (2000). Structural and rheological properties of methacrylamide modified gelatin hydrogels. Biomacromolecules.

[240] Engler AJ, Sen S, Sweeney HL, Discher DE (2006). Matrix elasticity directs stem cell lineage specification. Cell.

[241] Lee BH, Shirahama H, Cho N-J, Tan LP (2015). Efficient and controllable synthesis of highly substituted gelatin methacrylamide for mechanically stiff hydrogels. RSC Advances.

[242] Soofi SS, Last JA, Liliensiek SJ, Nealey PF, Murphy CJ (2009). The elastic modulus of Matrigel as determined by atomic force microscopy. Journal of Structural Biology.

[243] Mansour AA, Gonçalves JT, Bloyd CW, Li H, Fernandes S, Quang D (2018). An in vivo model of functional and vascularized human brain organoids. Nature Biotechnology.

[244] Cakir B, Xiang Y, Tanaka Y, Kural MH, Parent M, Kang Y-J (2019). Engineering of human brain organoids with a functional vascular-like system. Nature Methods.

[245] Shi Y, Sun L, Wang M, Liu J, Zhong S, Li R (2020). Vascularized human cortical organoids (vOrganoids) model cortical development in vivo. PLoS Biol.

[246] Song L, Yuan X, Jones Z, Griffin K, Zhou Y, Ma T (2019). Assembly of Human Stem Cell-Derived Cortical Spheroids and Vascular Spheroids to Model 3-D Brain-like Tissues. Science and Reports.

[247] Worsdorfer P, Dalda N, Kern A, Kruger S, Wagner N, Kwok CK (2019). Generation of complex human organoid models including vascular networks by incorporation of mesodermal progenitor cells. Science and Reports.

[248] Luissint AC, Artus C, Glacial F, Ganeshamoorthy K, Couraud PO (2012). Tight junctions at the blood brain barrier: Physiological architecture and disease-associated dysregulation. Fluids Barriers CNS.

[249] Uwamori H, Ono Y, Yamashita T, Arai K, Sudo R (2019). Comparison of organ-specific endothelial cells in terms of microvascular formation and endothelial barrier functions. Microvascular Research.

[250] Scherer HJ (1938). Structural Development in Gliomas. The American Journal of Cancer.

[251] Paredes I, Himmels P, Ruiz de Almodovar C (2018). Neurovascular Communication during CNS Development. Developmental Cell.

[252] Chang MY, Rhee YH, Yi SH, Lee SJ, Kim RK, Kim H (2014). Doxycycline enhances survival and self-renewal of human pluripotent stem cells. Stem Cell Reports.

[253] Cao D, Cheung HH, Chan WY (2019). Doxycycline Masks the Genuine Effect of the Doxycycline-Inducible Transgene by Promoting Dopaminergic Neuron Differentiation from Human Pluripotent Stem Cells. Stem Cells Dev.

[254] Lam D, Enright HA, Cadena J, Peters SKG, Sales AP, Osburn JJ (2019). Tissue-specific extracellular matrix accelerates the formation of neural networks and communities in a neuron-glia co-culture on a multi-electrode array. Scientific Reports.

[255] Petersen MA, Ryu JK, Akassoglou K (2018). Fibrinogen in neurological diseases: Mechanisms, imaging and therapeutics. Nature Reviews Neuroscience.

[256] Cho A-N, Jin Y, An Y, Kim J, Choi YS, Lee JS (2021). Microfluidic device with brain extracellular matrix promotes structural and functional maturation of human brain organoids. Nature Communications.

[257] Tang M, Rich JN, Chen S (2021). Biomaterials and 3D Bioprinting Strategies to Model Glioblastoma and the Blood-Brain Barrier. Advanced Materials.

[258] Wörsdörfer P, Rockel A, Alt Y, Kern A, Ergün S (2020). Generation of Vascularized Neural Organoids by Co-culturing with Mesodermal Progenitor Cells. STAR Protocols.

[259] Wimmer RA, Leopoldi A, Aichinger M, Kerjaschki D, Penninger JM (2019). Generation of blood vessel organoids from human pluripotent stem cells. Nature Protocols.

[260] Andersen J, Revah O, Miura Y, Thom N, Amin ND, Kelley KW (2020). Generation of Functional Human 3D Cortico-Motor Assembloids. Cell.

[261] Abelseth E, Abelseth L, De la Vega L, Beyer ST, Wadsworth SJ, Willerth SM (2019). 3D Printing of Neural Tissues Derived from Human Induced Pluripotent Stem Cells Using a Fibrin-Based Bioink. ACS Biomaterials Science & Engineering.

[262] Gu, Q., Tomaskovic-Crook, E., Wallace, G. G., & Crook, J. M. (2017). 3D bioprinting human induced pluripotent stem cell constructs for In Situ cell proliferation and successive multilineage differentiation. *Advanced Healthcare Materials, 6*(17), 1700175. 10.1002/adhm.201700175 10.1002/adhm.201700175 28544655

[263] Salaris, F., Colosi, C., Brighi, C., Soloperto, A., Turris, V., Benedetti, M. C., et al. (2019). 3D bioprinted human cortical neural constructs derived from induced pluripotent stem cells. *Journal of Clinical Medicine, 8*(10), 1595. 10.3390/jcm8101595 10.3390/jcm8101595 PMC6832547 31581732

[264] Salmon I, Grebenyuk S, Abdel Fattah AR, Rustandi G, Pilkington T, Verfaillie C (2022). Engineering neurovascular organoids with 3D printed microfluidic chips. Lab on a Chip.

